# Supplementary material for: Synthesis of Human Phase I and Phase II Metabolites of Hop (Humulus lupulus) Prenylated Flavonoids
Source: Metabolites. 2022 Apr 12;12(4):345. doi: 10.3390/metabo12040345 (PMC9030851; doi:10.3390/metabo12040345)
Supplement: Supplementary file 1 [file metabolites-12-00345-s001.zip › metabolites-1680284-supplementary.pdf]

Supplementary information

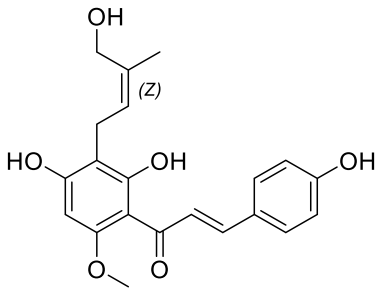

XN-OH (Z) 5.

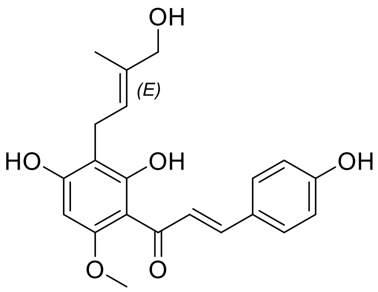

XN-OH (E) 6.

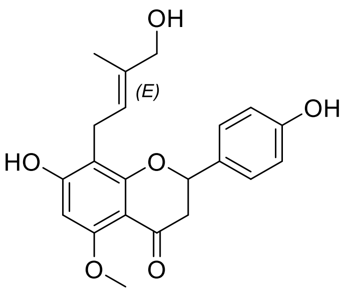

IXN-OH (E) 7.

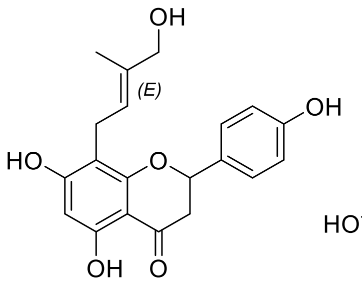

8-PN -OH (E) 8.

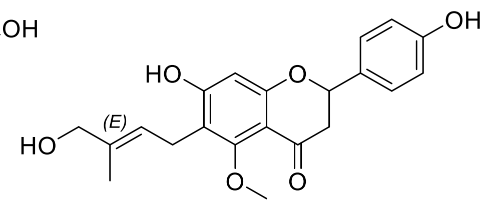

6PN-OH (E) 9.

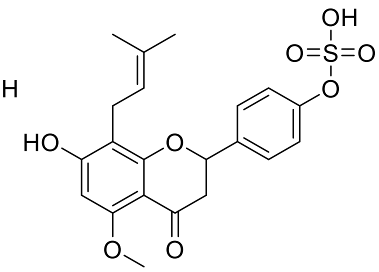

IXN-Sulf 10.

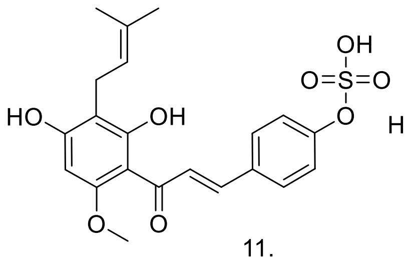

XN-Sulf 11.

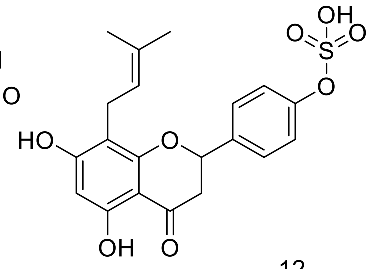

8-PN-Sulf 12.

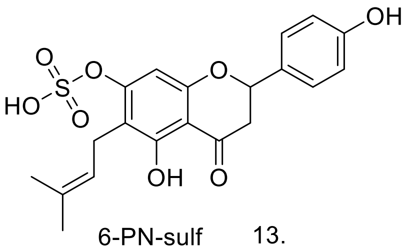

6-PN-sulf 13.

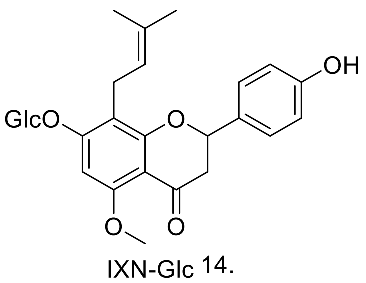

IXN-Glc 14.

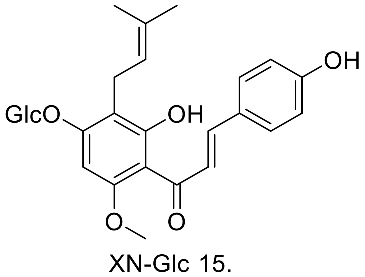

XN-Glc 15.

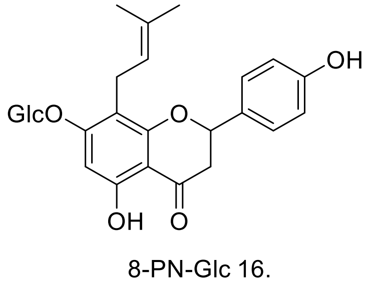

8-PN-Glc 16.

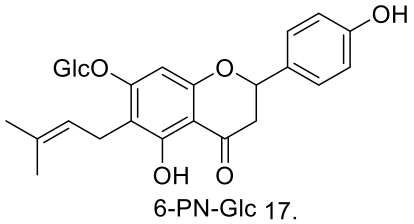

6-PN-Glc 17.

Figure S1: Structures of the newly synthesised compounds:

1. NMR and MS

Acetobromo-α-D-glucuronic acid methyl ester

ESI-: 338.90 (M+Br)

^{1}H NMR (500 MHz, $CDCl_3$) δ 6.64 (d, *J* = 4.0 Hz, 1H), 5.61 (t, *J* = 9.7 Hz, 1H, 6), 5.24 (dd, *J* = 10.3, 9.5 Hz, 1H), 4.85 (dd, *J* = 10.0, 4.1 Hz, 1H), 4.58 (d, *J* = 10.3 Hz, 1H), 3.76 (s, 3H, 22), 2.10 (s, 3H, 24), 2.05 (d, *J* = 3.1 Hz, 7H, 16, 20, 27), 1.29 – 1.22 (m, 1H), 0.07 (s, 1H). ^{13}C NMR (126 MHz, $CDCl_3$) δ 169.83, 169.63, 166.83, 85.47, 72.17, 70.46, 69.41, 68.62, 53.30, 29.85, 20.76, 20.61, 1.17. in accordance to jonkees et al. 2008 [1]

Compound 1, 2, 3, and 4

Xanthohumol, Isoxanthohumol, 6-Prenylnaringenin and 8-Prenylnaringenin were prepared previously [2].

Compound (5.) Xanthouhmol-Z-OH: (E)-1-(2,4-dihydroxy-3-((Z)-4-hydroxy-3-methylbut-2-en-1-yl)-6-methoxyphenyl)-3-(4-hydroxyphenyl)prop-2-en-1-one

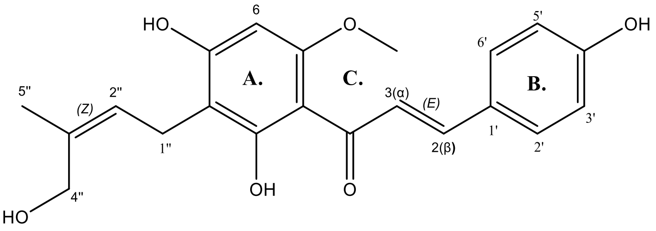

Figure S2: The structure of compound 5

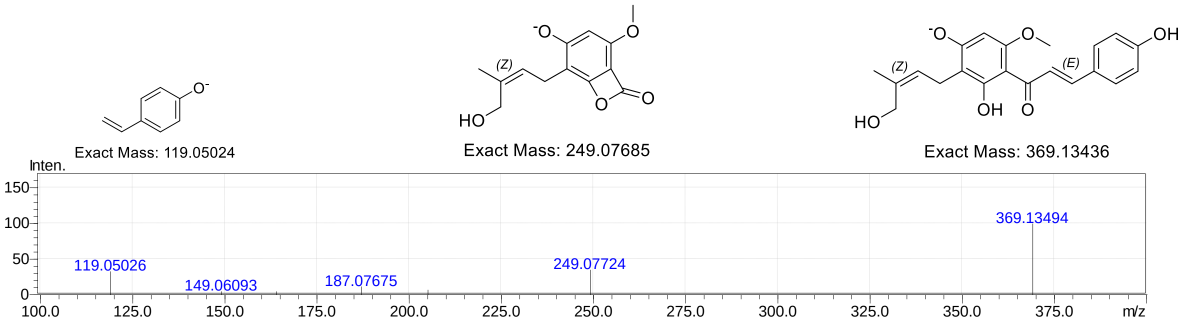

Figure S3: The MS/MS fragmentation of compound 5 and proposed structures.

^{1}H NMR (600 MHz, Acetone) δ 7.89 (d, *J* = 15.5 Hz, 1H, 3α), 7.74 (d, *J* = 15.6 Hz, 1H, 2β), 7.63 – 7.58 (m, 2H, 2', 6'), 6.94 – 6.89 (m, 2H, 3', 5'), 6.10 (s, 1H, 6), 5.34 (d, *J* = 8.6 Hz, 1H, 2''), 4.32 – 4.20 (m, 2H, 4''), 3.93 (s, 3H, OMe), 3.39 (d, *J* = 7.9 Hz, 2H, 1''), 1.72 (s, 3H, 5'').

^{13}C NMR (151 MHz, Acetone) δ 192.99 (4), 166.37 (8a), 164.10 (5), 162.16 (4'), 160.54 (7), 143.05 (2β), 135.06 (3''), 131.22 (2', 6'), 128.14 (3α), 126.14 (2''), 116.76 (3', 5'), 107.74 (4a), 105.95 (8), 92.35 (6), 61.96 (4''), 56.14 (OMe), 22.31 (1’’), 21.90 (5’’).

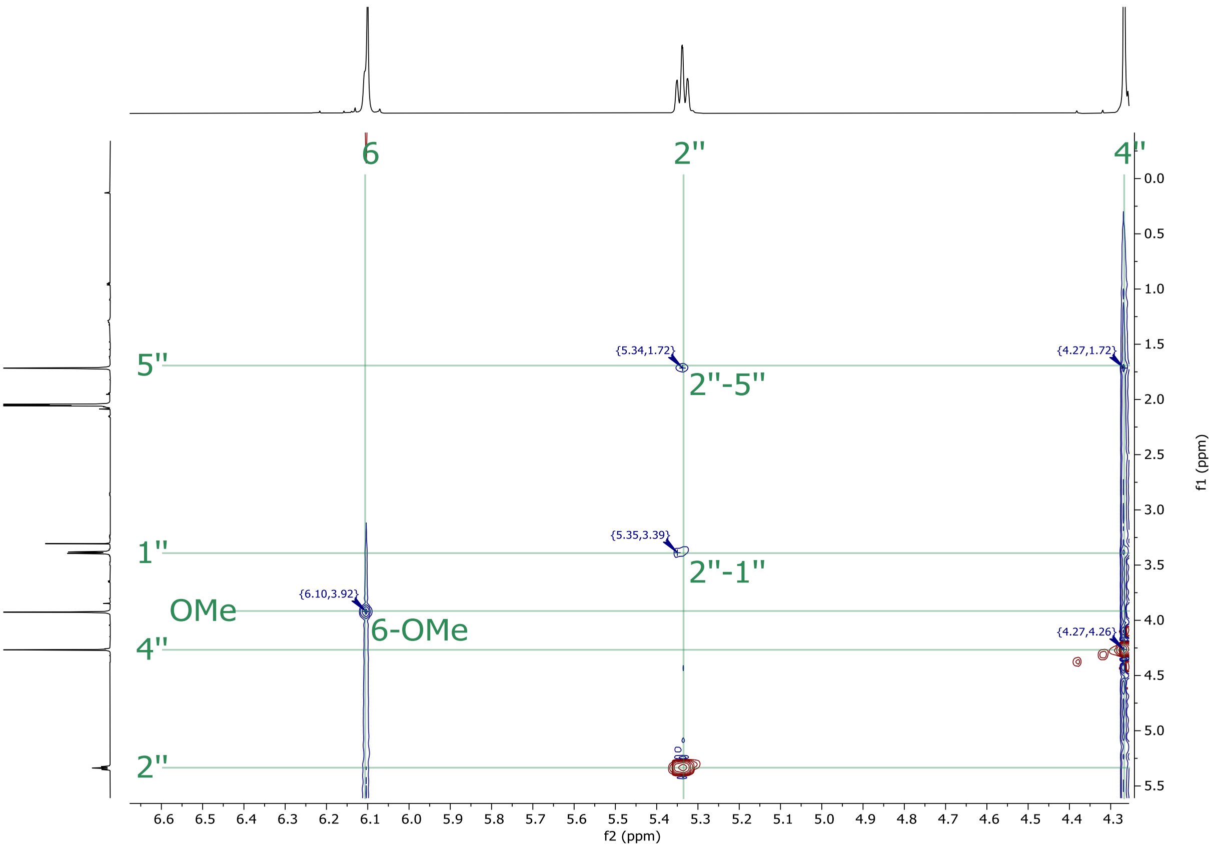

Figure S4: H-NOESY of XN-Z-OH shows the correlations between the free methyl group (5’’) and the 2’’ protons.

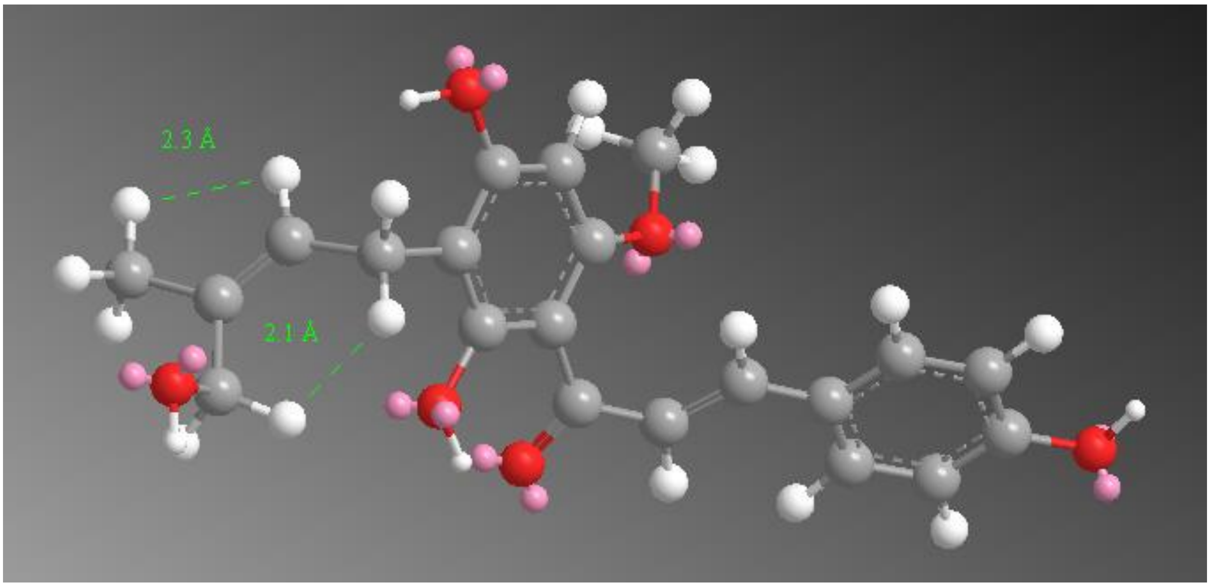

Figure S5: MM2 job minimisation after 1000 itreterations of XN-Z-OH. Showing the distances between the 2’’ and 5’’ protons of compound 5.

Compound (**6.**) Xanthouhmol-E-OH: (E)-1-(2,4-dihydroxy-3-((E)-4-hydroxy-3-methylbut-2-en-1-yl)-6-methoxyphenyl)-3-(4-hydroxyphenyl)prop-2-en-1-one

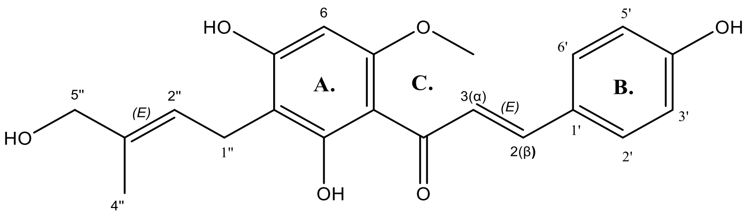

Figure S6: The strucuture of compound 6

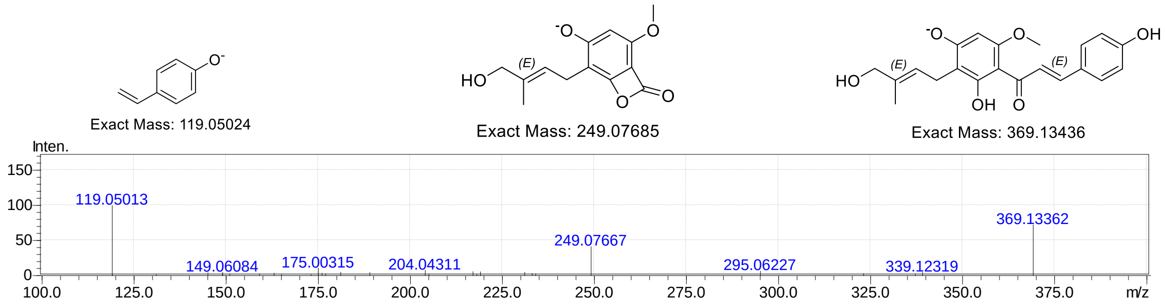

Figure S7: The MS/MS fragmentation of compound 6 and proposed structures

^{1}H NMR (600 MHz, Acetone) δ 7.88 (d, *J* = 15.5 Hz, 1H, 3α), 7.74 (d, *J* = 15.5 Hz, 1H, 2β), 7.63 – 7.58 (m, 2H, 2', 6'), 6.94 – 6.87 (m, 2H, 3', 5'), 6.16 (s, 1H, 6), 5.50 (t, *J* = 7.3 Hz, 1H, 2''), 3.92 (s, 3H, OCH3), 3.88 (s, 2H, 4''), 3.33 (d, *J* = 7.1 Hz, 2H, 1''), 1.78 (s, 3H, 5'').

^{13}C NMR (151 MHz, Acetone) δ 193.18 (4), 193.15, 166.46 (7), 165.86 (5), 162.00 (8a), 160.58 (4'), 142.99 (2β), 135.60 (3''), 131.24 (2', 6'), 128.10 (3α), 125.44 (1'), 116.75 (3', 5'), 108.56 (s, 8), 106.15 (4a)d, 91.78 (6), 68.43 (4''), 56.14 (OCH3), 21.61 (1''), 13.82 (5’’).

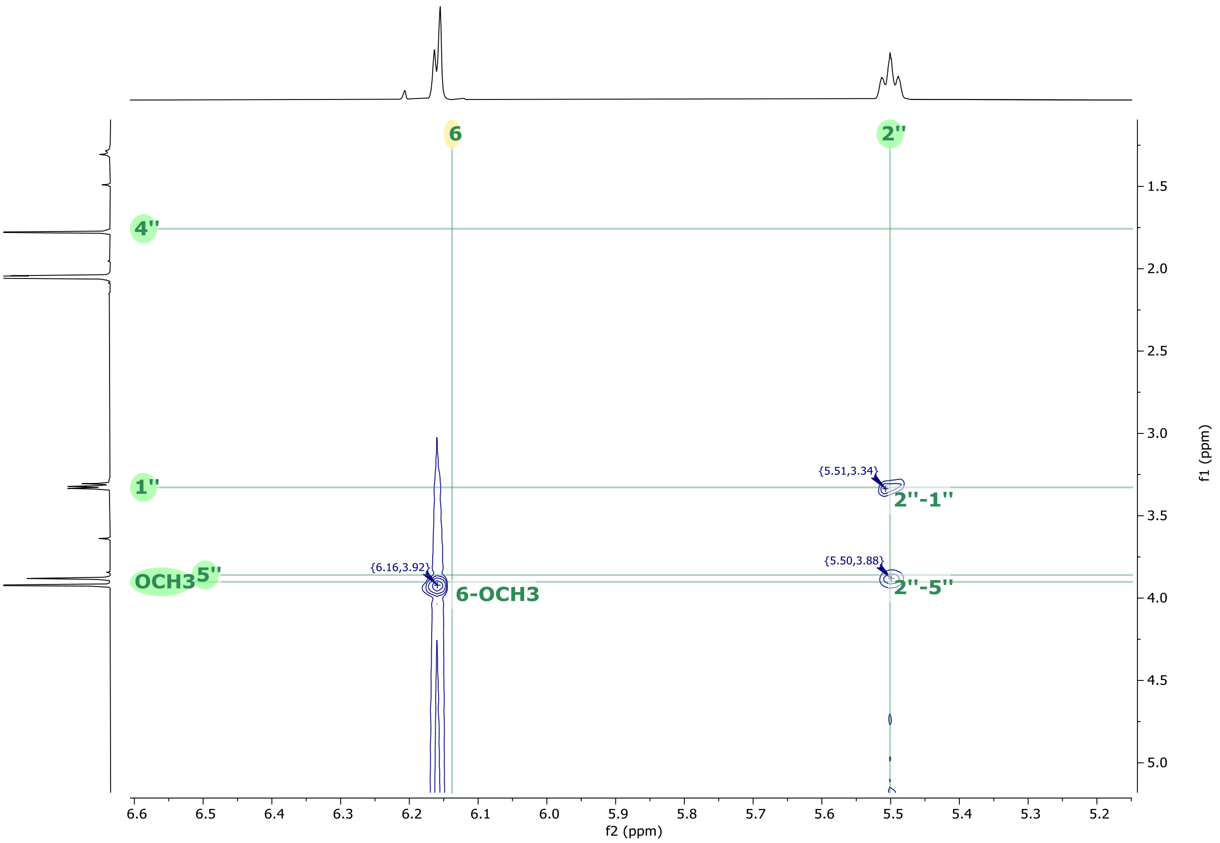

Figure S8: The ^{1}H-NOESY of compound 6

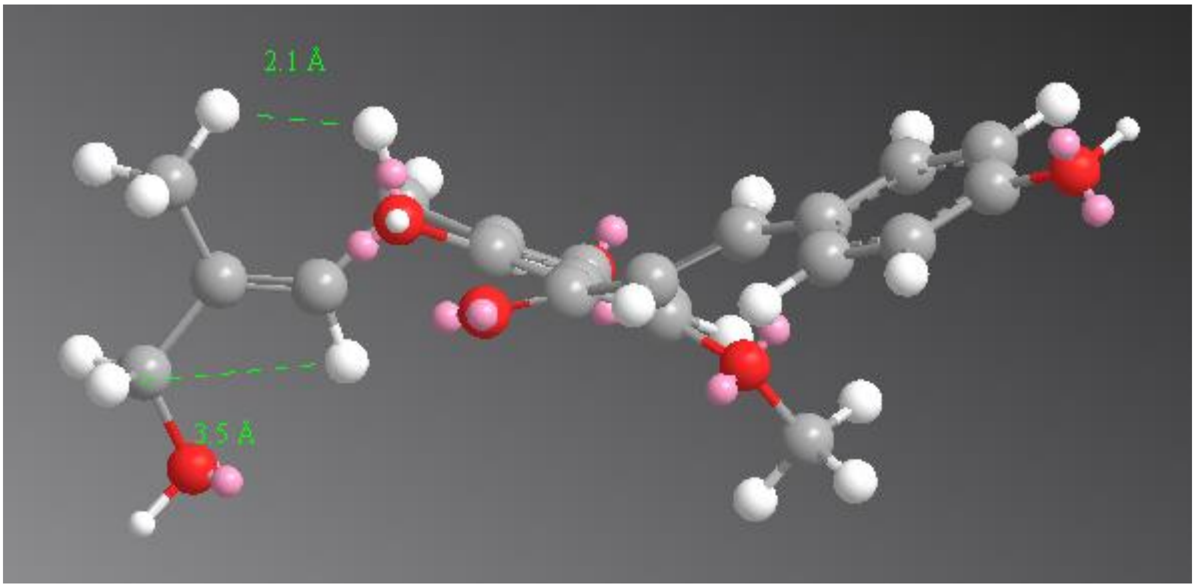

Figure S9: MM2 job minimisation after 1000 itreterations of XN-*Z*-OH. Showing the distances between the 2'' and 5'' protons of compound 6.

Compound (**7.**) Isoxanthohumol-E-OH: (E)-7-hydroxy-8-(4-hydroxy-3-methylbut-2-en-1-yl)-2-(4-hydroxyphenyl)-5-methoxychroman-4-one

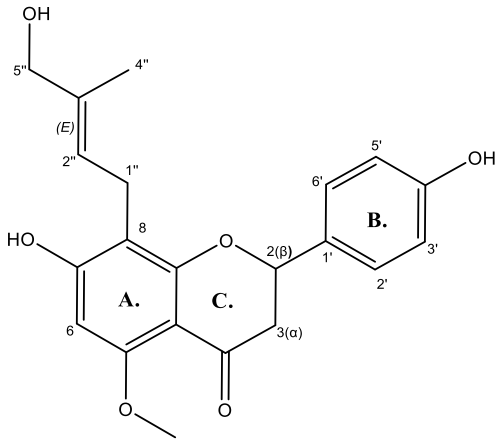

Figure S10: The strucuture of compound 7

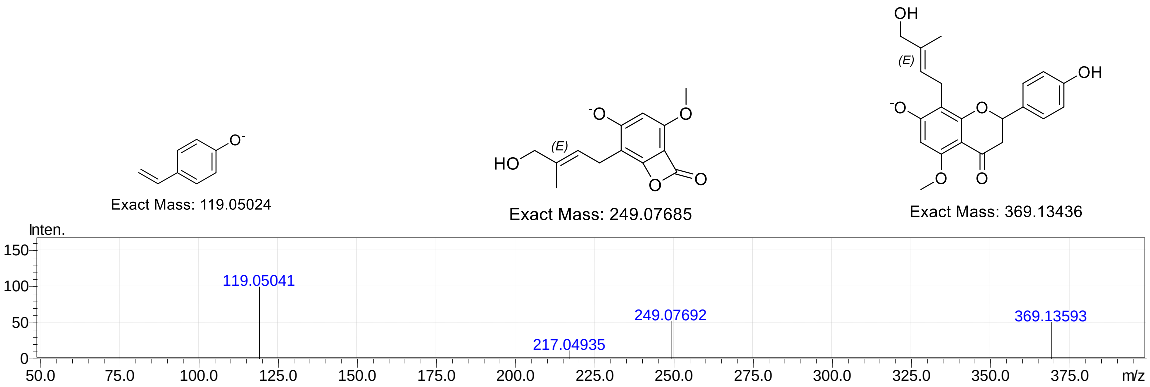

Figure S11: The MS/MS fragmentation of compound 7 and proposed structures

^{1}H NMR (600 MHz, Acetone) δ 7.39 (d, *J* = 8.5 Hz, 2H, 2', 6'), 6.89 (d, *J* = 8.5 Hz, 2H, 3', 5'), 6.23 (s, 1H, 6), 5.50 – 5.42 (m, 1H, 2''), 5.35 (d, *J* = 12.8 Hz, 1H, 2β), 3.87 – 3.83 (m, 2H, 5''), 3.74 (s, 3H, OCH3), 3.33 – 3.26 (m, 2H, 1''), 2.97 – 2.87 (m, 1H, 3α''), 2.65 – 2.57 (m, 1H, 3α'), 1.62 (s, 3H, 4'').

^{13}C NMR (126 MHz, Acetone) δ 187.81 (4), 162.02 (8a), 161.31 (7), 160.34 (5), 157.48 (4'), 134.80 (3''), 130.64 (1'), 127.84 (2', 6'), 122.57 (2''), 115.16 (3', 5'), 107.78 (8), 105.32 (4a), 92.77 (6), 78.65 (2β), 67.47 (5''), 54.98 (OCH3), 45.34 (3α), 21.17 (1''), 12.95 (4'').

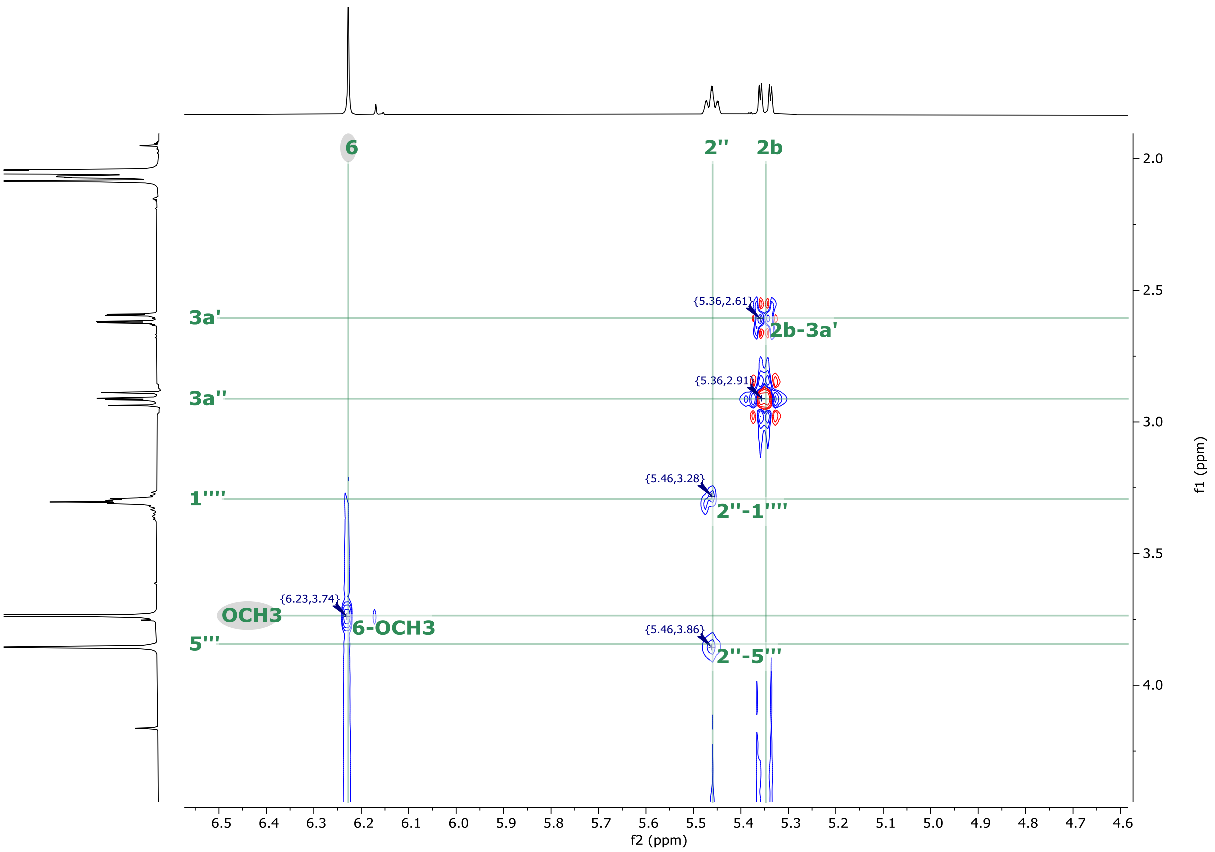

Figure S12: The ^{1}H-NOESY of compound 7

Compound (8.) 8-Prenylnaringenin-E-OH : (E)-5,7-dihydroxy-8-(4-hydroxy-3-methylbut-2-en-1-yl)-2-(4-hydroxyphenyl)chroman-4-one

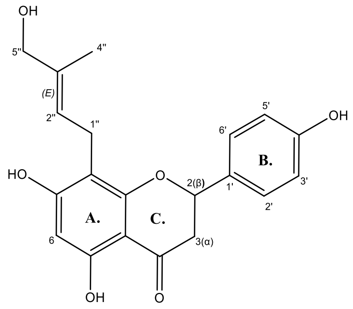

Figure S13: The structure of compound 8

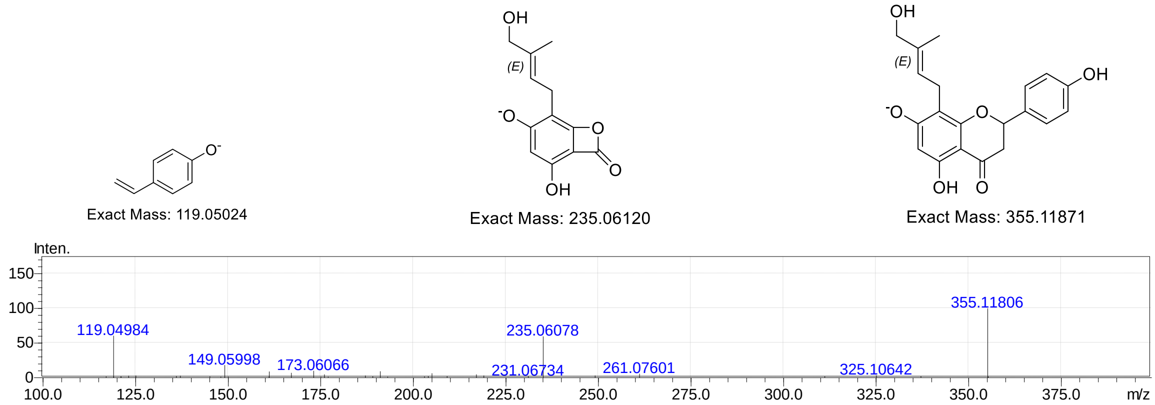

Figure S14: The MS/MS fragmentation of compound 8 and proposed structures

^{1}H NMR (400 MHz, Acetone) δ 7.41 (d, *J* = 8.5 Hz, 2H, 1', 2'), 6.90 (d, *J* = 8.5 Hz, 2H, 3', 6'), 6.04 (s, 1H, 6), 5.45 (m, 1H, 2β), 5.42 (m, 1H, 2''), 3.86 (s 2H, 5''), 3.26 (d, *J* = 7.2 Hz, 2H, 1''), 3.12 (m, 1H, 3α''), 2.75 (m,1H 3α'), 1.62 (s, 3H, 4'').

^{13}C NMR (101 MHz, Acetone) δ 197.37 (4), 165.51 (8a), 163.09 (7), 161.12 (5), 158.56 (4'), 135.75 (3''), 131.11 (1), 128.88 (1', 2'), 123.36 (2''), 116.13 (3', 6'), 108.06 (8), 103.12 (4a), 96.46 (6), 79.73 (2β), 68.38 (5''), 43.47 (3α), 21.81 (1''), 13.82 (4'').

Compound (9.) 6-Prenylnaringenin-E-OH: (E)-5,7-dihydroxy-6-(4-hydroxy-3-methylbut-2-en-1-yl)-2-(4-hydroxyphenyl)chroman-4-one

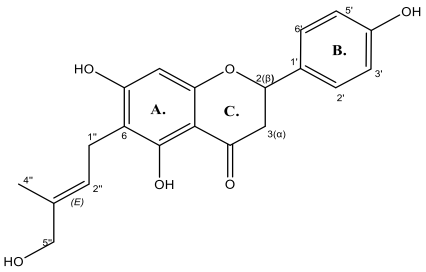

Figure S15: The structure of compound 9

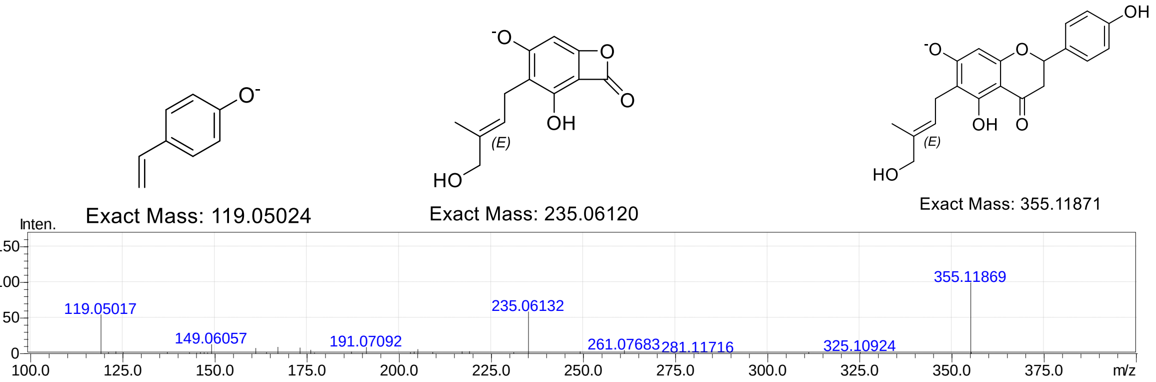

Figure S16: The MS/MS fragmentation of compound 9 and proposed structures

^{1}H NMR (500 MHz, Acetone) δ 7.51 – 7.29 (m, 2H, 2', 6'), 6.92 – 6.85 (m, 2H, 3', 5'), 6.04 (s, 1H, 8), 5.49 (t, *J* = 8.7, 7.3, 1.5 Hz, 1H, 2β), 5.45 – 5.36 (m, 1H, 2''), 3.88 (s, 2H, 5''), 3.29 (d, *J* = 7.3 Hz, 2H, 1''), 3.20 – 3.08 (m, 1H, 3α''), 2.71 (ddd, *J* = 17.1, 7.7, 3.1 Hz, 1H, 3α'), 1.77 (s, 3H, 4'').

^{3}C NMR (126 MHz, Acetone) δ 197.21 (4), 165.28 , (8a), 162.18 (5, 7), 158.65 (4'), 135.81 (3''), 130.94 (1'), 129.00 ,(2', 6'), 123.25 (2''), 116.13 ,(3', 5') 108.73 ,(6) 102.98 (8), 95.41 (4a), 79.84 (2β), 68.38 (5''), 43.59 , (3α), 21.17 (1''), 13.78 (4'').

Compound (10.) Isoxanthohumol-4’-O-sulfate: 4-(7-hydroxy-5-methoxy-8-(3-methylbut-2-en-1-yl)-4-oxochroman-2-yl)phenyl hydrogen sulfate

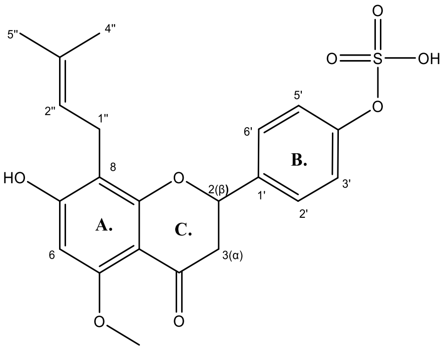

Figure S17: The structure of compound 10

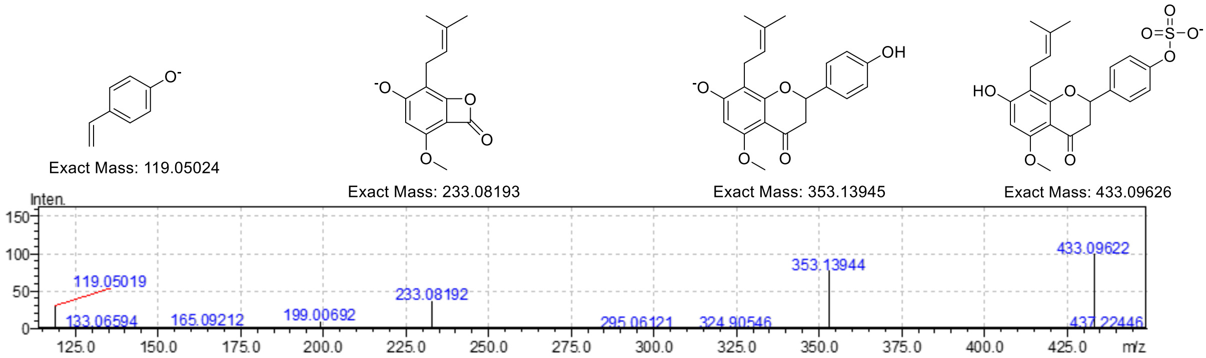

Figure S18: The MS/MS fragmentation of compound 10 and proposed structures

^{1}H NMR (400 MHz, MeOD) δ 7.51 – 7.45 (m, 2H, 2', 6'), 7.39 – 7.31 (m, 2H, 3', 5'), 6.13 (s, 1H, 6), 5.40 (dd, *J* = 12.6, 3.2 Hz, 1H, 2β), 5.15 (ddt, *J* = 8.6, 7.2, 1.4 Hz, 1H, 2''), 3.80 (s, 3H, OMe), 3.27 – 3.19 (m, 2H, 1''), 2.96 (dd, *J* = 16.7, 12.6 Hz, 1H, 3α''), 2.73 (dd, *J* = 16.7, 3.2 Hz, 1H, 3α'), 1.61 (d, *J* = 12.0 Hz, 6H, 4'', 5''),

^{13}C NMR (126 MHz, MeOD) δ 196.31 (4), 164.72 (8a), 161.79 (7), 159.86 (5) 154.05 (1') 137.17 (4'), 131.76 (3’’), 128.17 (2', 6'), 123.94 (2''), 122.54 (3', 5'), 110.08 (8), 105.78 (4a), 93.65 (6), 79.62 (2β), 55.96 (OMe), 46.32 (3α), 25.95 (4’’), 22.70 (1’’), 17.96 (5'').

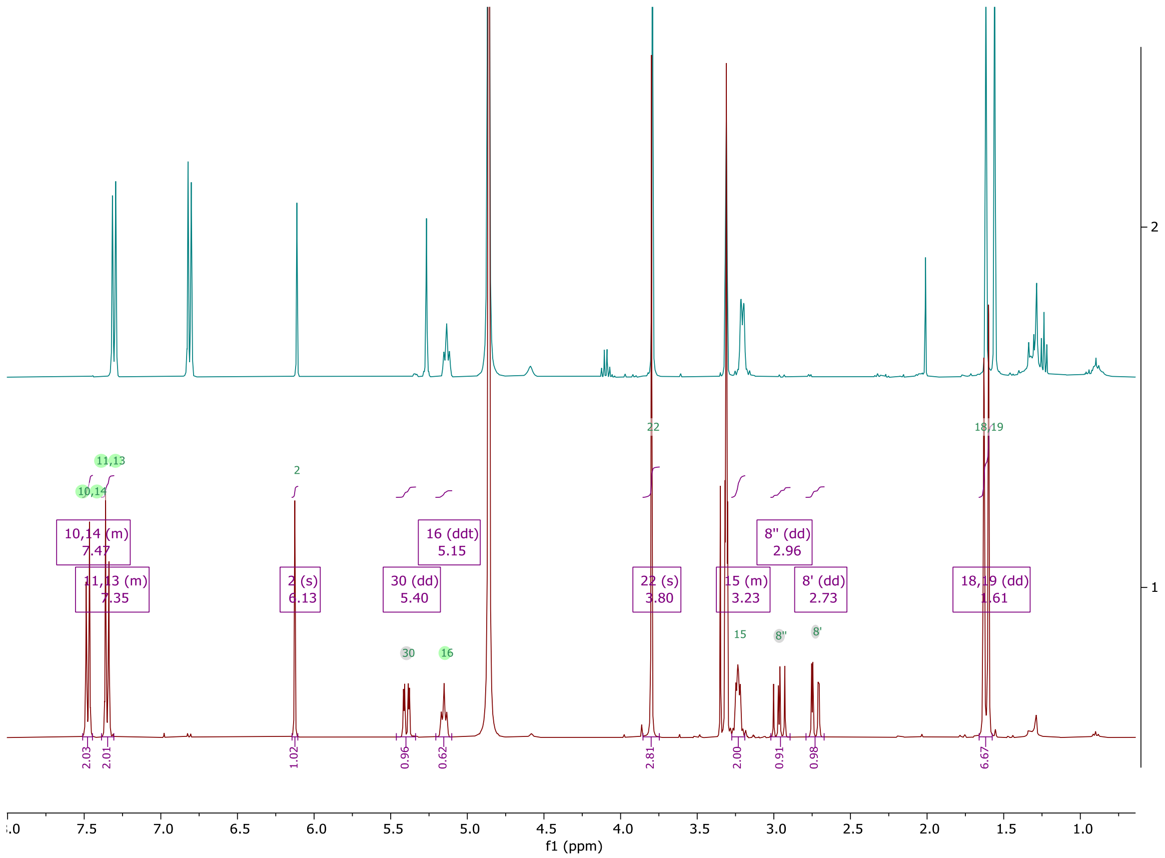

Figure S19: H1 spectrum of (Blue) IXN stacked against (red) IXN-4’-O-sulfate

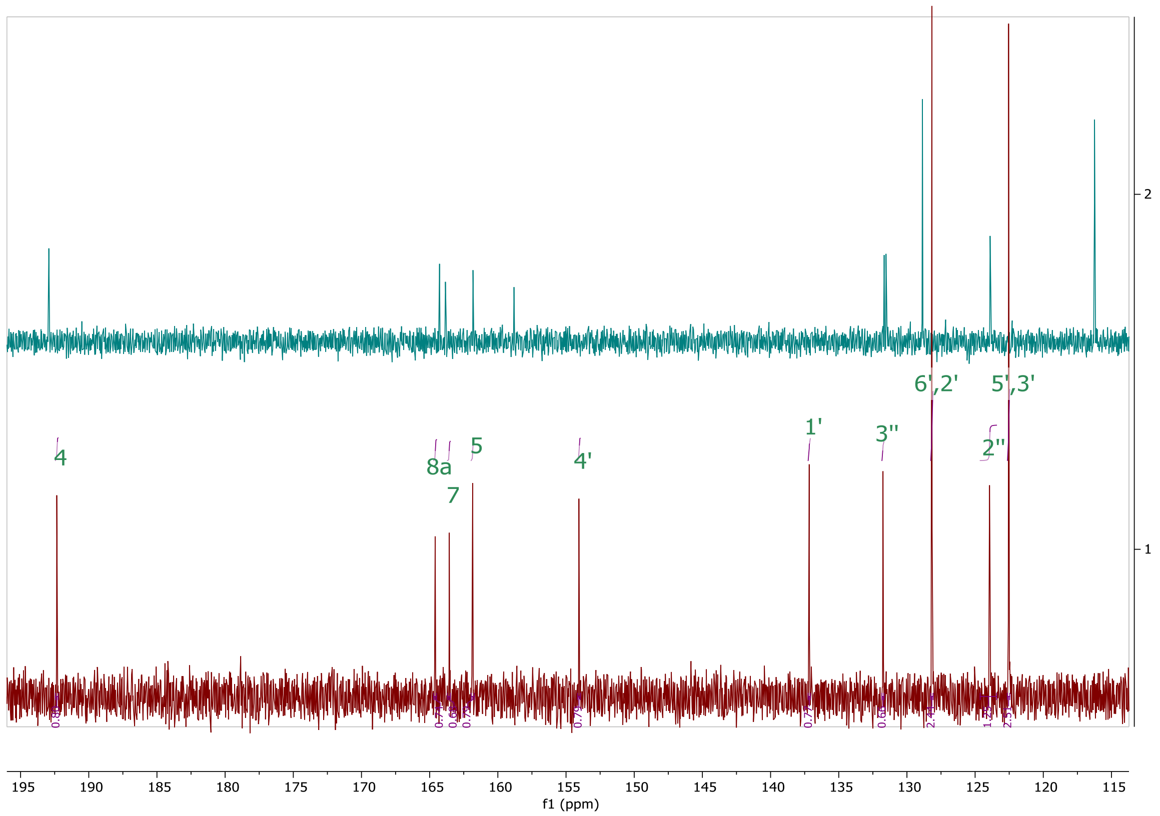

Figure S20: A zoomed in area of the carbon spectra of IXN (cyan) and IXN-4'O-sulfate (red)

To figure out the position of the sulfate group the chemical shift of that carbon should be significantly shifted upfield therefore, we looked at position 4' and carbon 7 as significant changes should occur at either of these positions. As C7 is relatively unchanged and position 4' and the 5',3' have moved to significantly (158 towards 153 and 116 towards 122 ppm repectively) ppms indicating that the sulfate is conjugated on ring B – see Figure 20.

Compound (11.): Xanthohumol-4’-O-Sulfate: (E)-4-(3-(2,4-dihydroxy-6-methoxy-3-(3-methylbut-2-en-1-yl)phenyl)-3-oxoprop-1-en-1-yl)phenyl hydrogen sulfate

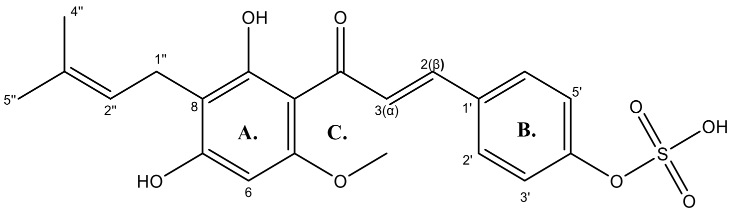

Figure S21: The structure of compound 11

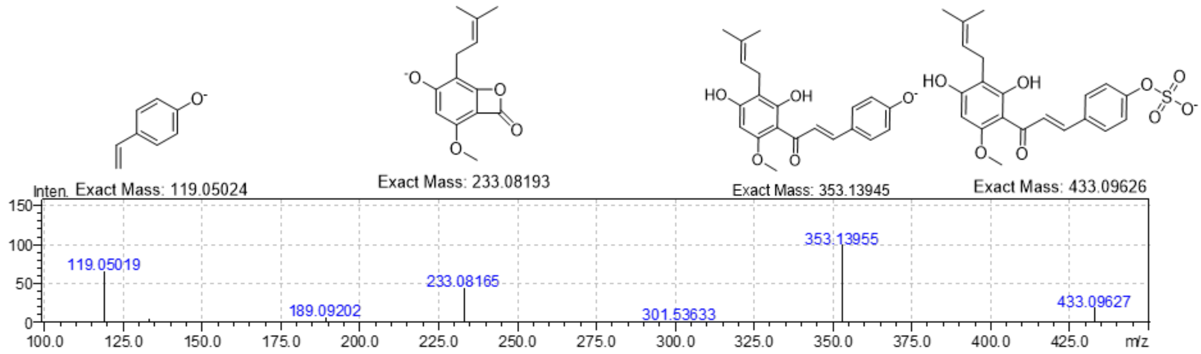

Figure S22: The MS/MS fragmentation of compound 11 and proposed structures

^{1}H NMR (500 MHz, MeOD) δ 7.89 (d, *J* = 15.6 Hz, 1H, 3α), 7.68 (d, *J* = 15.6 Hz, 1H, 2β), 7.65 – 7.61 (m, 2H, 2', 6'), 7.39 – 7.33 (m, 2H, 3', 5'), 6.03 (s, 1H, 6), 5.21 (tt, J = 7.3, 4.3, 1.5 Hz, 1H, 2''), 3.91 (s, 3H, OMe), 3.23 (d, *J* = 7.1 Hz, 2H, 1'',), 1.76 (s, 3H, 4'',), 1.65 (s, 3H, 5'')

^{13}C NMR (126 MHz, MeOD) δ 193.90 , 4, 166.22 , 8a, 164.05 , 7, 162.53 , 5, 155.58 , 4', 141.91 , 2a, 133.41 , 1', 131.35 , 3'', 130.27 , 2', 6', 128.63 , 3b, 124.21 , 2'', 122.68 , 3', 5', 109.37 , 8, 106.47 , 4a, 91.62 , 6, 56.21 , OMe, 25.98 , 4'', 5'', 22.26 , 1'', 17.88.

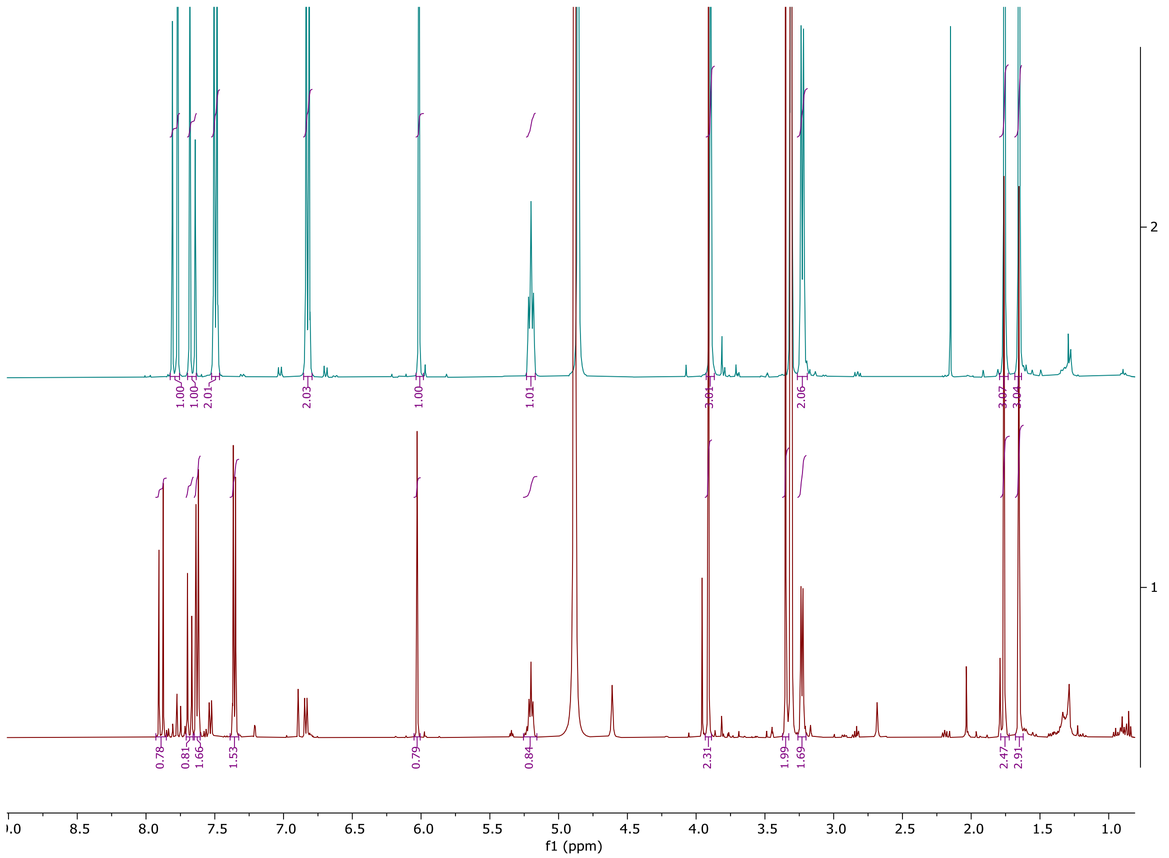

Figure S23: H1 spectrum of (Blue) XN stacked against (red) XN-4’O-sulfate

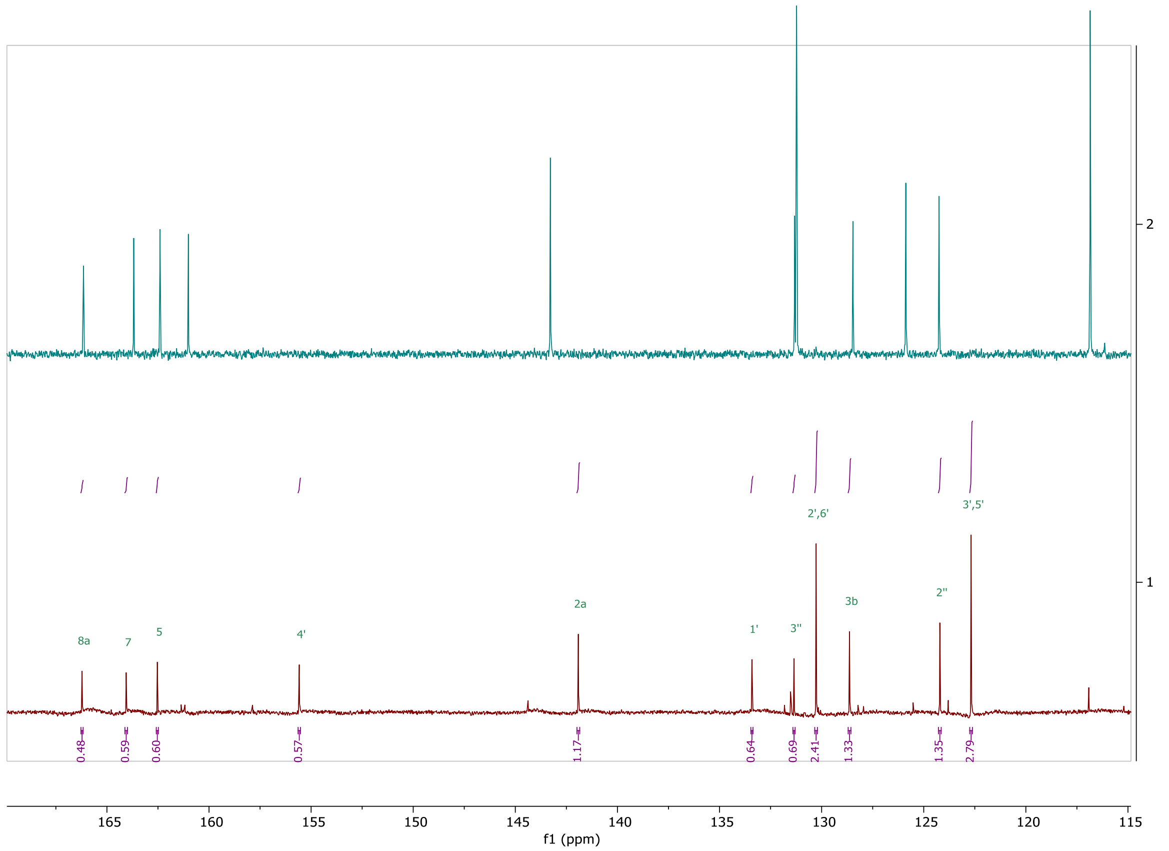

Figure S24: A zoomed in area of the carbon spectra of XN (cyan) and XN-4’O-sulfate (red)

To figure out the position of the sulfate group the chemical shift of that carbon should be significantly shifted upfield therefore, we looked at position 4’ and carbon 7 as significant changes should occur at either of these positions. As C7 is relatively unchanged and position 4’ and the 5’,3’ have moved to significantly (161 towards 155 and 116 towards 122 ppm repectively) ppms indicating that the sulfate is conjugated on ring B – See Figure 24.

Compound (12.): 8-Prenylnaringenin-4’O sulfate: 4-(5,7-dihydroxy-8-(3-methylbut-2-en-1-yl)-4-oxochroman-2-yl)phenyl hydrogen sulfate

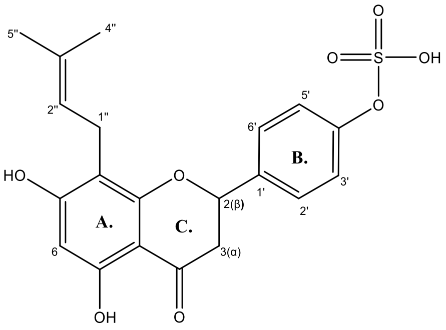

Figure S25: The structure of compound 12

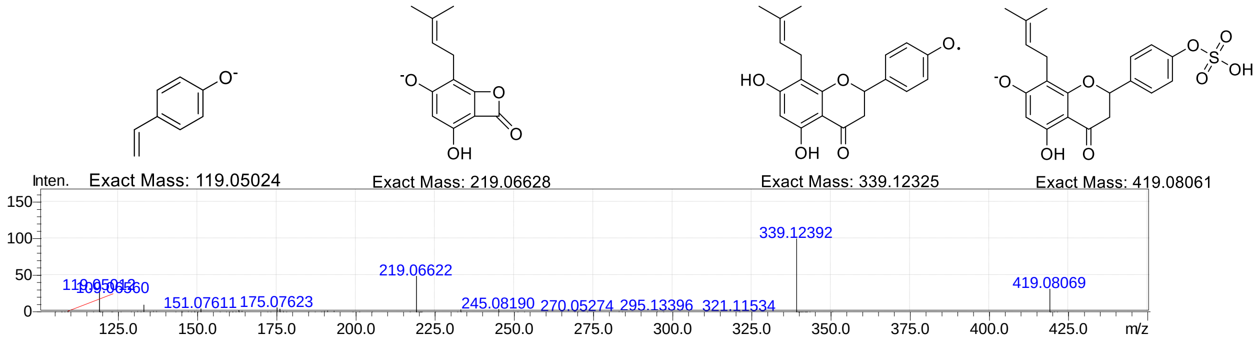

Figure S26: The MS/MS fragmentation of compound 12 and proposed structures

^{1}H NMR (500 MHz, MeOD) δ 7.53 – 7.46 (m, 2H, 2', 6'), 7.39 – 7.29 (m, 2H, 3', 5'), 5.94 (s, 1H, 6), 5.44 (dd, *J* = 12.5, 3.1 Hz, 1H), 5.15 (t, *J* = 7.2, 1.4 Hz, 1H), 3.26 – 3.13 (m, 2H), 3.17 (p, *J* = 1.7 Hz, 0H, 1''), 3.07 (dd, *J* = 17.1, 12.5 Hz, 1H, 3a), 2.79 (dd, *J* = 17.1, 3.2 Hz, 1H), 1.63 (s, 3H), 1.60 (s, 3H, 4'', 5'').

^{13}C NMR (126 MHz, MeOD) δ 196.31 (4), 164.72 (8a) 161.79 (7) 159.86 (5), 152.68 (4'), 135.67 (3''), 130.29 (1’), 126.87 (2', 6'), 122.53 (2''), 121.18 (3', 5'), 107.69 (8), 101.96 ,(4a), 95.08 (6), 78.45 (2a), 42.67 (3a), 24.56 (5’’), 21.07 (1''), 16.54 (4'').

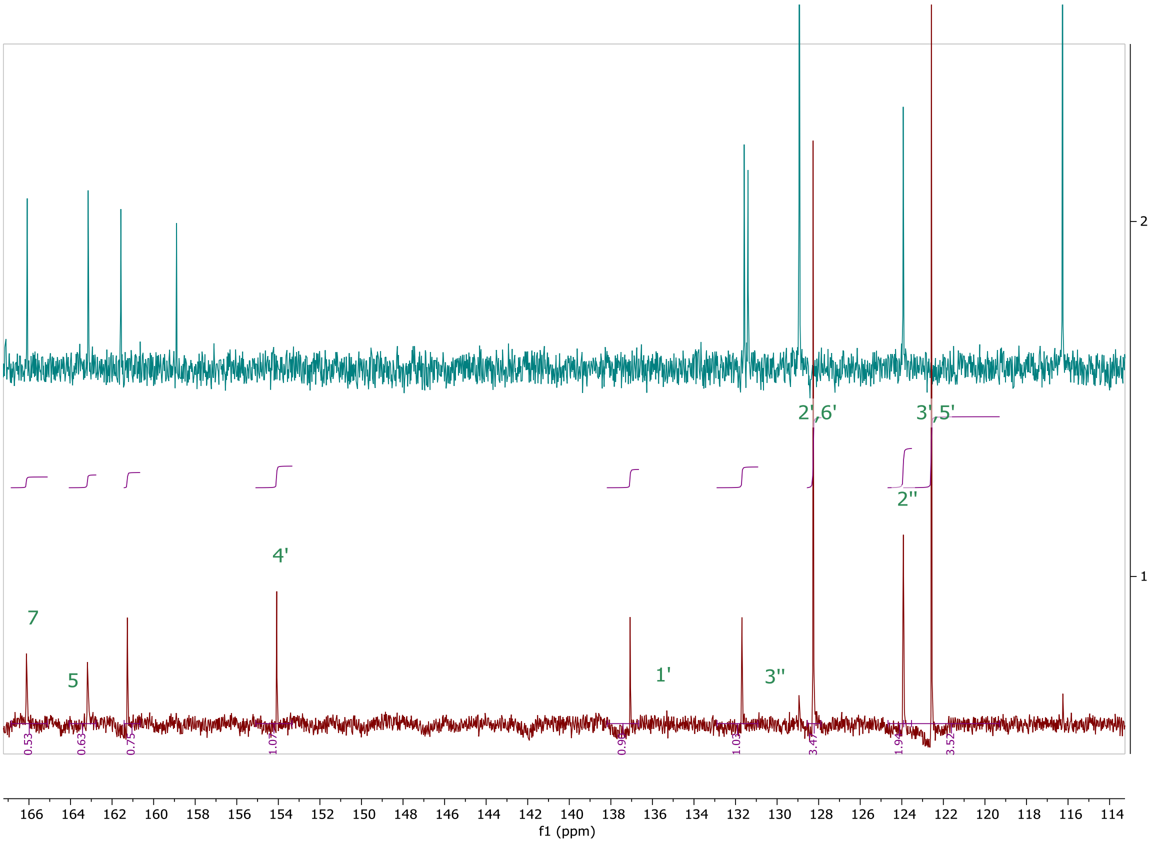

Figure S27: A zoomed in area of the carbon spectra of 8PN (cyan) and 8PN-4'O-sulfate (red)

To figure out the position of the sulfate group the chemical shift of that carbon should be significantly shifted upfield therefore, we looked at position 4' and carbon 7 as significant changes should occur at either of these positions. As C7 is relatively unchanged and position 4' and the 5',3' have moved to significantly (159 towards 154 and 116 towards 122 ppm repectively) ppms indicating that the sulfate is conjugated on ring B See Figure 27.

Compound (13.): 6-Prenylnaringenin-7-O-sulfate: 5-hydroxy-2-(4-hydroxyphenyl)-6-(3-methylbut-2-en-1-yl)-4-oxochroman-7-yl hydrogen sulfate

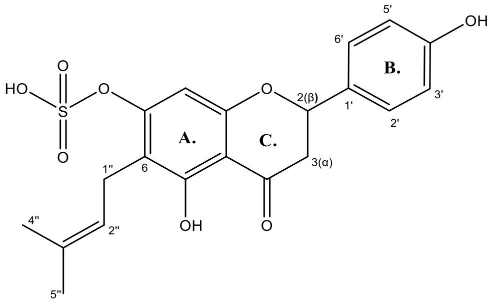

Figure S28: The structure of compound 13

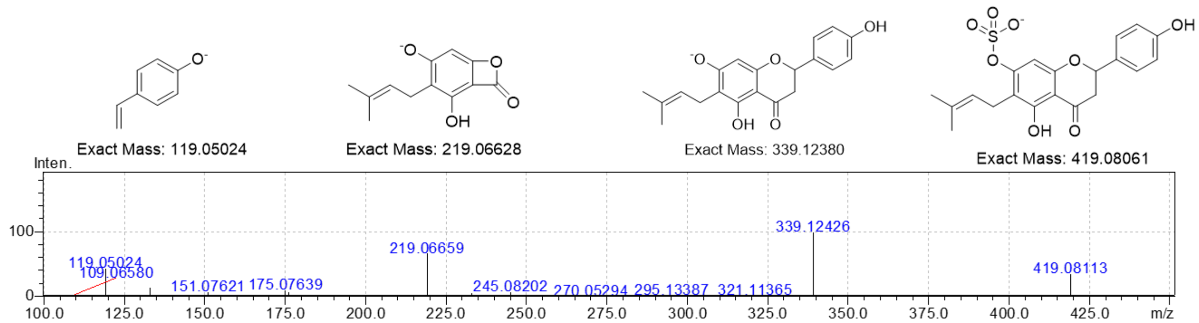

Figure S29: The MS/MS fragmentation of compound 13 and proposed structures

^{1}H NMR (500 MHz, MeOD) δ 7.30 (d, *J* = 8.7 Hz, 2H, 2', 6'), 6.81 (d, *J* = 8.7 Hz, 2H, 3', 5'), 5.90 (s, 1H, 8), 5.28 (dd, *J* = 13.0, 2.9 Hz, 1H, 2''), 5.19 (t, *J* = 7.2 Hz, 1H, 2a), 3.20 (d, *J* = 7.2 Hz, 2H, 1''), 3.07 (dd, *J* = 17.1, 13.0 Hz, 1H, 3b'), 2.65 (dd, *J* = 17.1, 3.1 Hz, 1H, 3b''), 1.75 (s, 3H, 4'',), 1.65 (s, 3H. 5'').

^{13}C NMR (126 MHz, MeOD) δ 197.21 (4), 168.01 (8a), 162.58 (7), 162.43(5) , 158.96 (4'), 131.36 (3''), 131.35 (1'), 128.98 (3', 5'), 124.18 (2''), 116.29 (2', 6'), 109.95 (6), 102.72 (4a), 96.06 (8), 80.33 (2a), 44.16 , 3b, 25.96 (5’’), 21.92 (1''), 17.87 (4'').

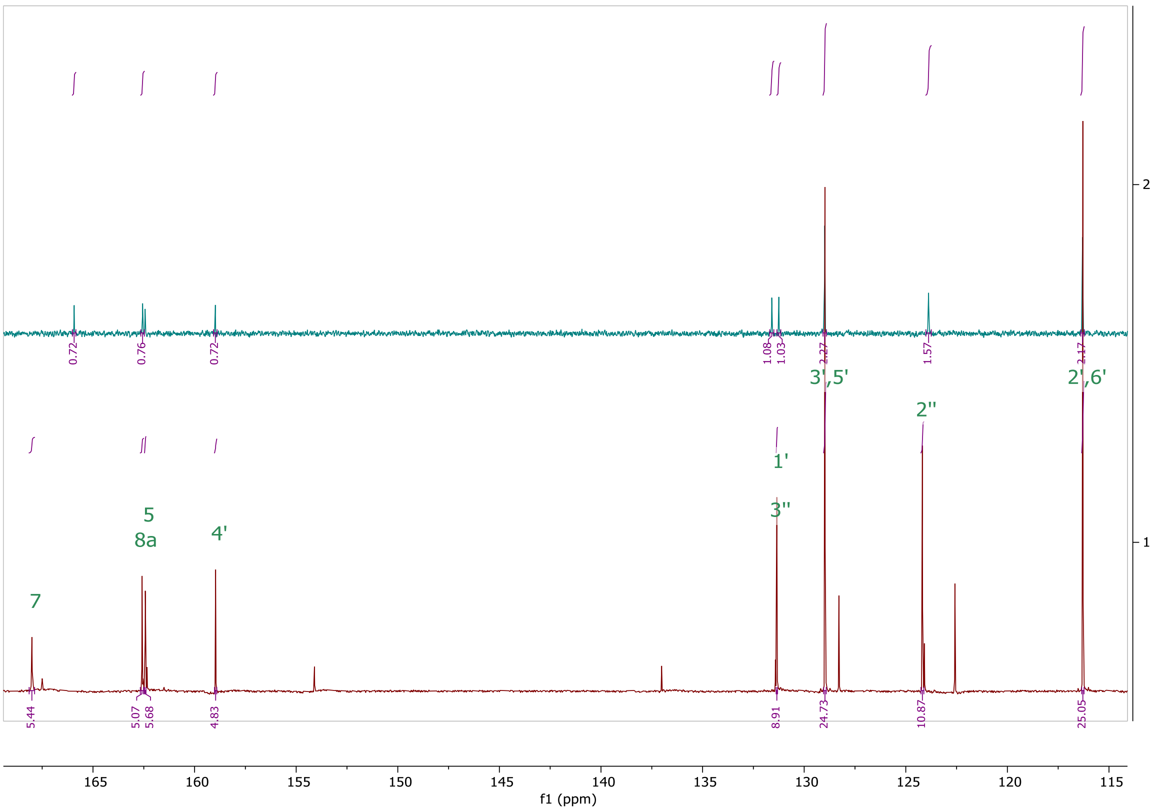

Figure S30: A zoomed in area of the carbon spectra of 6PN (cyan) and 6PN-7-O-sulfate (red)

To find out the position of the sulfate group the chemical shift of that carbon should be significantly shifted to higher PPM near where the sulfate is conjugated therefore, we looked at position 4’ and carbon 7. Differing from the other sulfates the 2’,6’ and 3’,5’ signals have not moved, but position 7

has changed from 165 towards 168 indicating that the sulfate is conjugated to this position. See figure 30.

Compound (14.): IXN 7-O-Glucuronide: (2S,3S,4S,5R,6S)-3,4,5-trihydroxy-6-((2-(4-hydroxyphenyl)-5-methoxy-8-(3-methylbut-2-en-1-yl)-4-oxochroman-7-yl)oxy)tetrahydro-2H-pyran-2-carboxylate

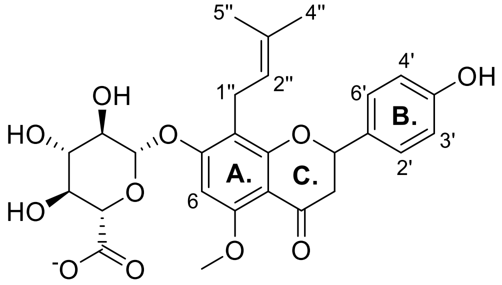

Figure S31 The structure of compound 14

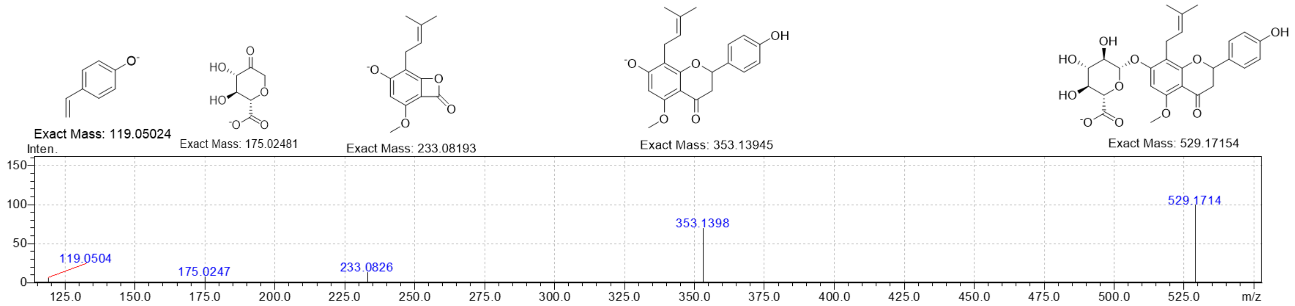

Figure S32: The MS/MS fragmentation of compound 14 and proposed structures

^{1}H NMR (500 MHz, MeOD) δ 7.34 – 7.26 (m, 2H, 2', 6'), 6.86 – 6.78 (m, 2H, 3', 5'), 6.55 (s, 1H, 6), 5.32 (t, *J* = 12.8, 9.8, 2.9 Hz, 1H, 2a), 5.19 – 5.15 (m, 1H, 2''), 5.07 (dd, *J* = 14.2, 7.0 Hz, 1H, 9), 3.86 (s, 2H, OMe), 3.71 – 3.43 (m, 2H, 10, 11, 12), 3.27 – 3.14 (m, 1H, 13), 3.03 (ddd, *J* = 16.7, 12.7, 9.5 Hz, 1H, 3b''), 2.76 – 2.66 (m, 1H, 3b'), 1.58 (d, 6H).

^{13}C NMR (126 MHz, MeOD) δ 193.19 (4), 163.22 (8a), 163.14 (7), 163.01 (5), 161.68 (4'), 158.90 (14), 131.77 (3''), 131.30 (1'), 128.96 (2', 6'), 123.89 (2''), 116.23 (3', 5'), 112.73 (8), 107.67 (4a), 102.03 (9), 93.82 (6), 80.17 (2a), 78.09 (13), 74.74 (10, 11, 12), 56.28 (OMe), 46.30 (3b), 25.95 (5''), 22.93 (1''), 18.05 (4'').

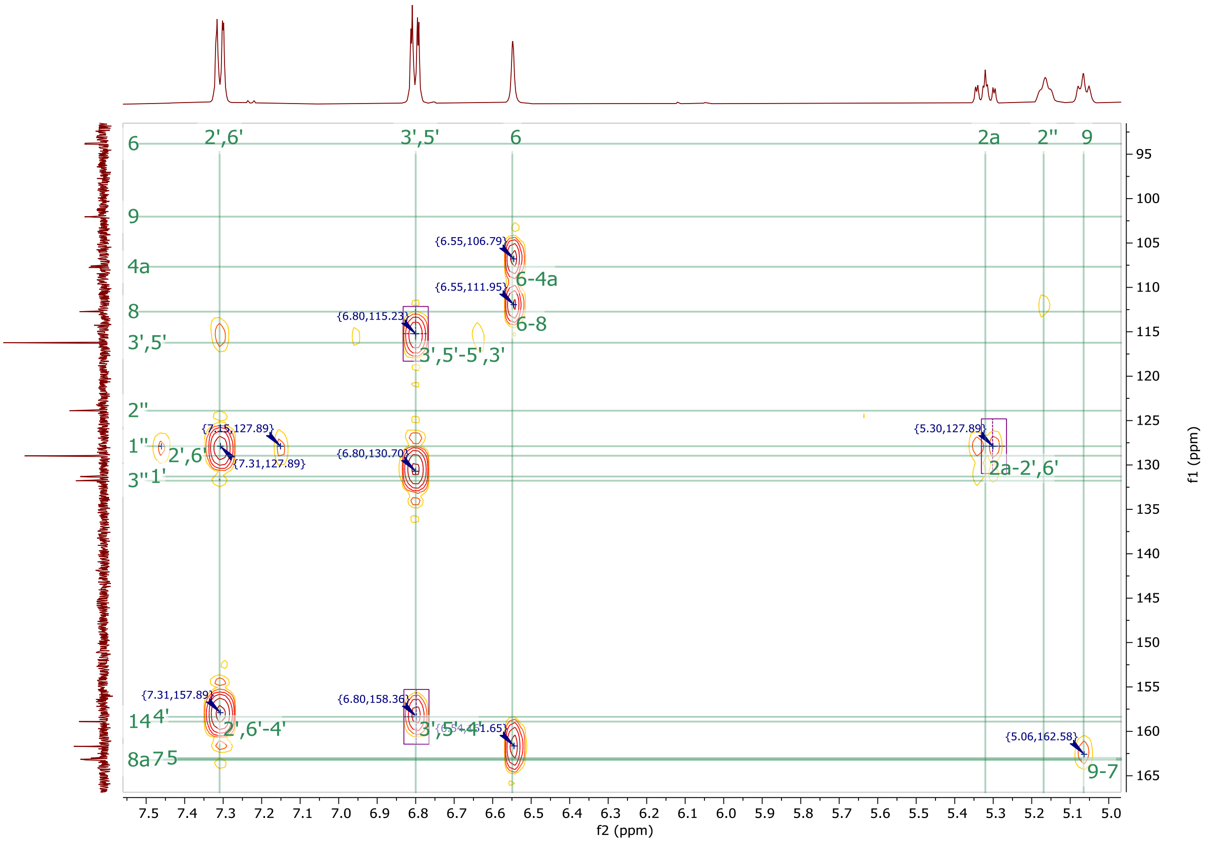

Figure S33: HMBC of IXN-7-O-glc

Indications that the glucuronide is attached to the 7 position is that there is a correlation between the anomeric proton of the glucuronide and the C7 as indicated in figure 33.

Compound (15.): XN 7-O-Glucuronide: (2S,3S,4S,5R,6S)-3,4,5-trihydroxy-6-(3-hydroxy-4-((E)-3-(4-hydroxyphenyl)acryloyl)-5-methoxy-2-(3-methylbut-2-en-1-yl)phenoxy)tetrahydro-2H-pyran-2-carboxylate

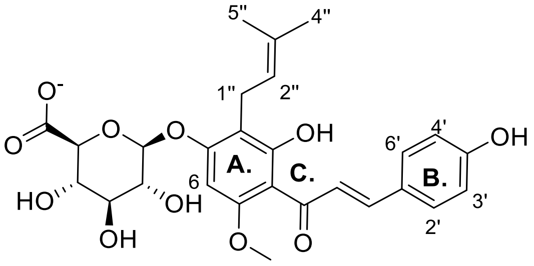

Figure S34: The structure of compound 15

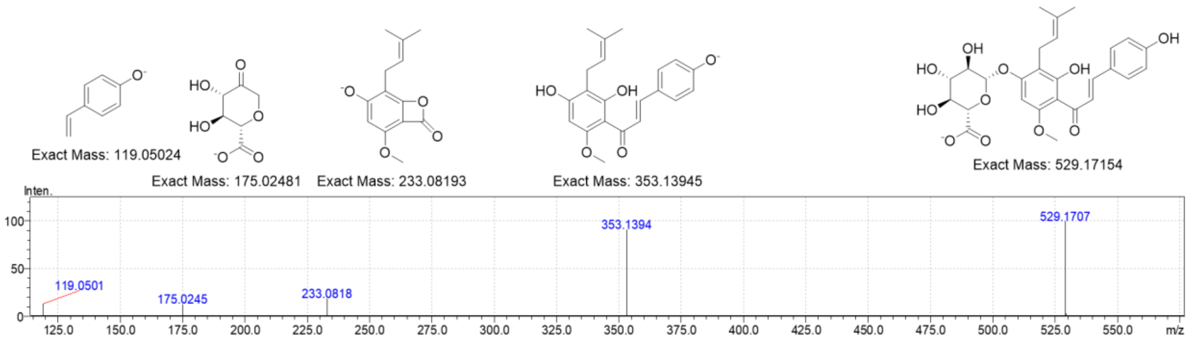

Figure S35: The MS/MS fragmentation of compound 15 and proposed structures

^{1}H NMR (600 MHz, MeOD) δ 7.78 (d, *J* = 15.5 Hz, 1H, 3b), 7.71 (d, *J* = 15.5 Hz, 1H), 7.58 – 7.47 (m, 2H, 2', 6'), 6.90 – 6.80 (m, 2H, 3', 5'), 6.40 (s, 1H, 6), 5.23 (tt, *J* = 7.2, 1.5 Hz, 1H, 2''), 5.13 (d, *J* = 7.5 Hz, 1H, 10), 4.03 (d, *J* = 9.7 Hz, 1H, 14), 3.95 (s, 3H, OMe), 3.76 – 3.64 (m, 1H, 13), 3.62 – 3.50 (m, 2H, 11, 12), 3.40 (dd, *J* = 14.1, 7.5 Hz, 1H, 1''), 3.30 – 3.26 (m, 1H), 1.72 (dd, *J* = 80.7, 1.6 Hz, 6H, 4'', 5'').

^{13}C NMR (151 MHz, MeOD) δ 194.83 (4) , 164.94 (8a), 162.30 (7), 162.17 (5), 161.28 (4'), 144.17 (2a), 131.62 (3''), 131.45 (2', 6'), 128.28 (1'), 125.56 (3b), 124.12 (2''), 116.91 (3', 5'), 112.54 (8), 108.49 (4a), 102.04 (10), 91.70 (6), 77.74 (12), 76.55 (14), 74.67 (11), 72.72 (13), 56.42 (OMe), 25.95 (5''), 22.54 (1''), 18.06 (4'').

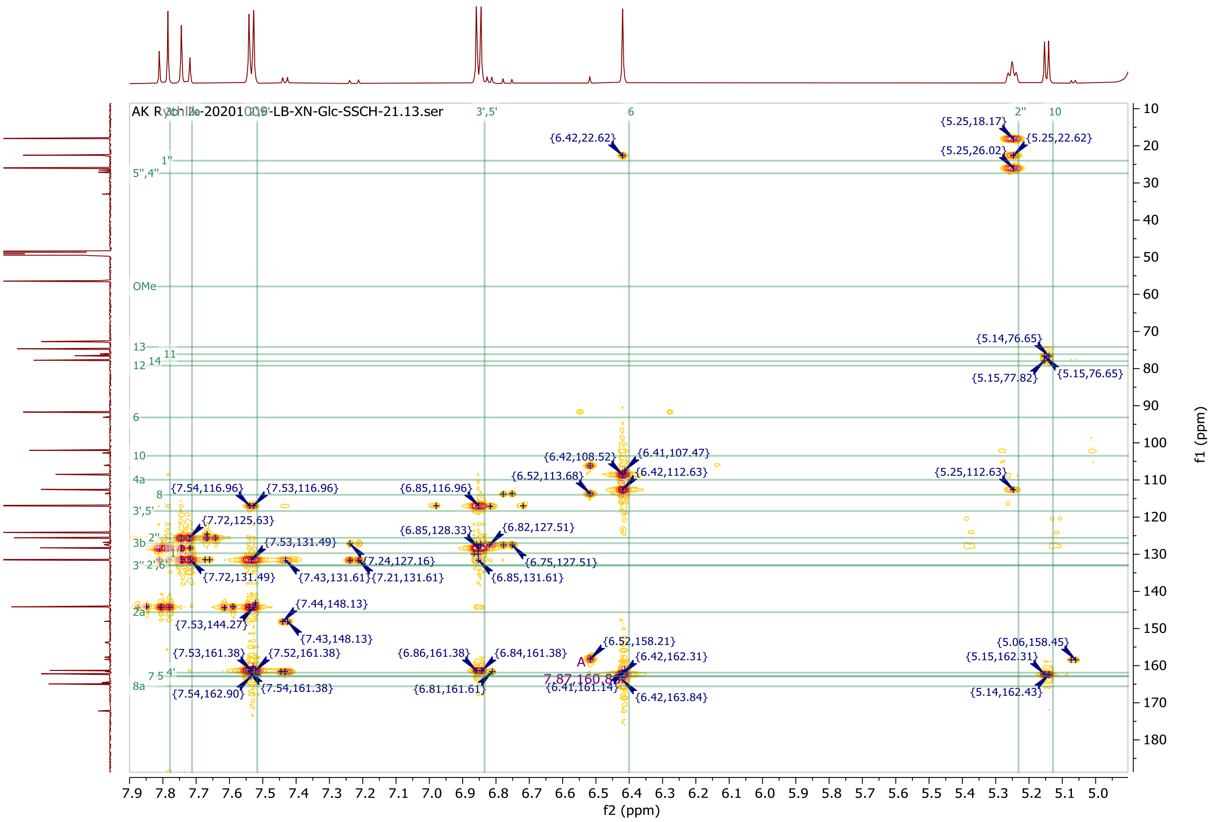

Figure S36: HMBC of XN-7-O-glc

Indications that the glucuronide is attached to the 7 position is that there is a correlation between the anomeric proton of the glucuronide and the C7 as indicated in figure 36

Compound (16.): 8-Prenylnaringenin-7-O-Glucuronide: (2S,3S,4S,5R,6S)-3,4,5-trihydroxy-6-((5-hydroxy-2-(4-hydroxyphenyl)-8-(3-methylbut-2-en-1-yl)-4-oxochroman-7-yl)oxy)tetrahydro-2H-pyran-2-carboxylate

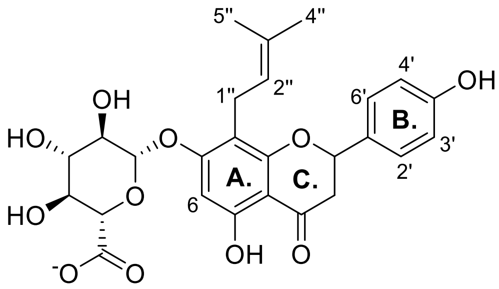

Figure S37: The structure of compound 16

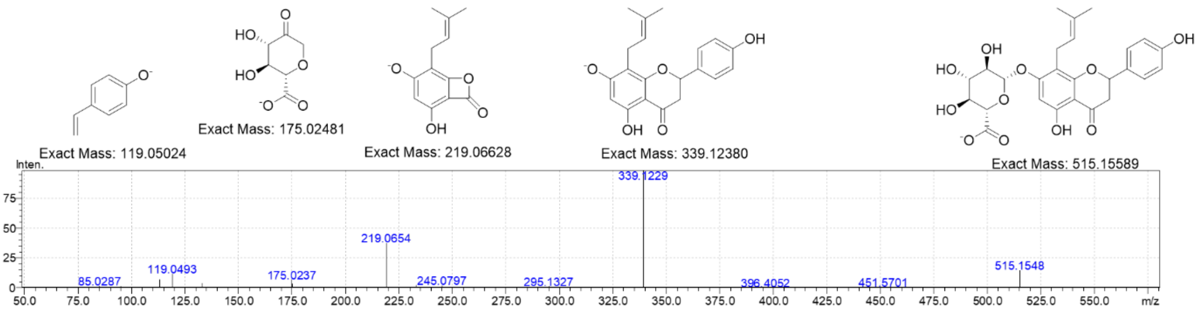

Figure S38: The MS/MS fragmentation of compound 16 and proposed structures

^{1}H NMR (600 MHz, MeOD) δ 7.37 – 7.29 (m, 2H, 2', 6'), 6.86 – 6.78 (m, 2H, 3', 5'), 6.27 (s, 1H, 6), 5.37 (t, *J* = 13.1, 3.0 Hz, 1H, 2), 5.19 (t, 1H, 2''), 5.07 (d, *J* = 10.9, 7.4 Hz, 1H, 9), 4.01 – 3.94 (m, 1H), 3.64 – 3.47 (m, 2H, 10, 11, 12, 13), 3.35 (s, 1H), 3.39 – 3.28 (m, 0H), 3.28 – 3.10 (m, 2H, 3''), 2.77 (ddd, *J* = 17.1, 14.2, 3.1 Hz, 1H, 3'), 1.61 (s, 3H), 1.57 (s, 3H).

^{13}C NMR (151 MHz, MeOD) δ 198.84 , 4, 176.82 , 14, 164.51 , 8a, 164.39 , 7, 162.75 , 5, 161.57, 159.04 , 4', 149.74, 140.64, 131.78 , 3'', 131.04 , 1', 129.09, 129.07, 123.84 (d, *J* = 3.3 Hz, 2''), 116.29 , 3', 5', 111.86 , 8, 104.73 , 4a, 101.32 , 9, 95.29 , 6, 80.66 , 2, 80.62 , 13, 77.91 , 11, 74.63 , 10, 73.18 , 12, 44.32 , 3, 25.94, 24.60 , 4'', 22.09 , 1'', 18.01.

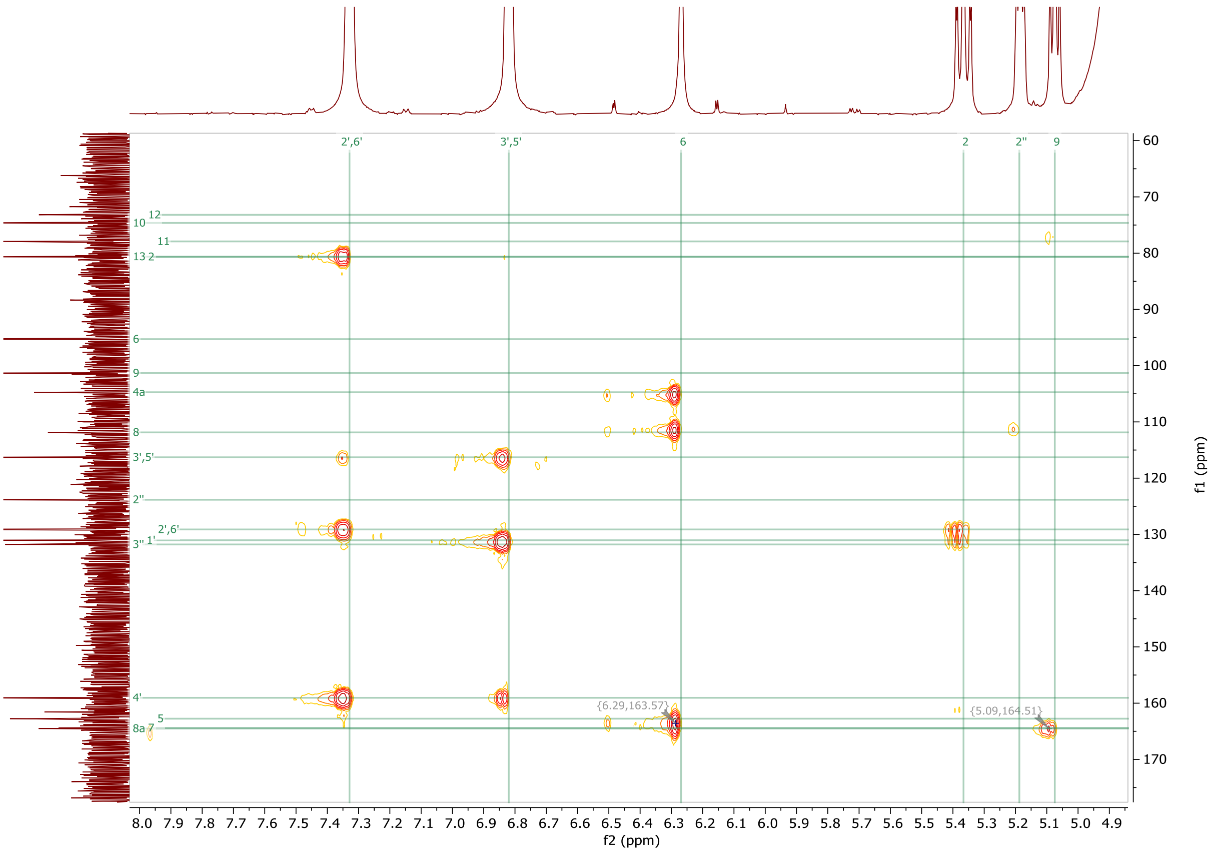

Figure S39: HMBC of 8-PN-7-O-glc

Indications that the glucuronide is attached to the 7 position is that there is a correlation between the anomeric proton of the glucuronide and the C7 as indicated in figure 39

Compound (17.): 6-Prenylnaringenin-7-O-glucuronide: (2S,3S,4S,5R,6S)-3,4,5-trihydroxy-6-((5-hydroxy-2-(4-hydroxyphenyl)-6-(3-methylbut-2-en-1-yl)-4-oxochroman-7-yl)oxy)tetrahydro-2H-pyran-2-carboxylate

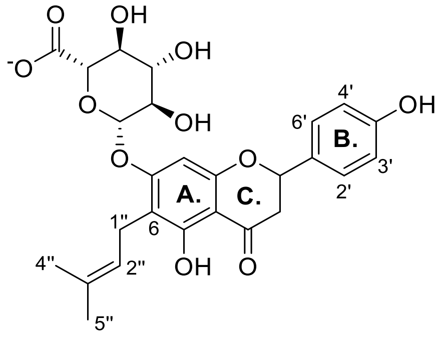

Figure S40: The structure of compound 17

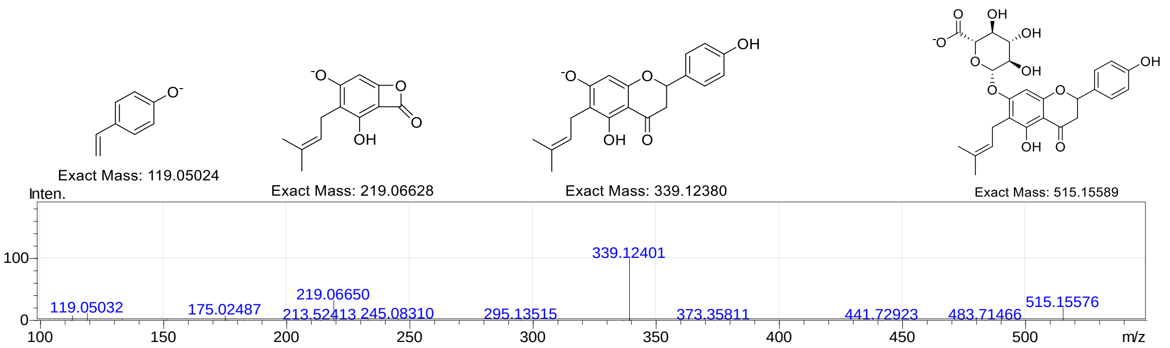

Figure S41: The MS/MS fragmentation of compound 17 and proposed structures

^{1}H NMR (500 MHz, MeOD) δ 7.33 (dd, J = 8.6, 2.8 Hz, 2H, 2, 6), 6.82 (dd, J = 8.6, 2.7 Hz, 2H), 6.27 (s, 1H, 13), 5.37 (td, J = 12.1, 3.0 Hz, 1H, 19), 5.18 (t, J = 7.4 Hz, 1H, 34), 5.11 – 5.04 (m, 1H, 23), 3.98 (dd, J = 9.8, 4.3 Hz, 1H, 20), 3.58 – 3.47 (m, 1H, 21, 22, 25), 3.35 (d, 0H, 33''), 3.27 – 3.10 (m, 1H, 9), 2.82 – 2.72 (m, 1H), 1.59 (d, J = 21.8 Hz, 7H).

^{13}C NMR (126 MHz, MeOD) δ 199.03 , 4, 164.42 , 8a, 163.08 , 7, 163.01, 161.05 , 5, 158.99 , 4', 131.78 , 3'', 131.12 , 1', 129.02 , 2', 6', 123.85 , 2'', 116.28 , 3', 5', 111.26 , 6, 105.00 , 4a, 101.42 , 9, 96.33 , 8, 80.46 , 2, 77.71, 76.63 , 13, 74.57 , 11, 72.99 , 10, 44.22, 43.98, 25.94 , 4'', 5'', 22.69 , 1'', 18.04.

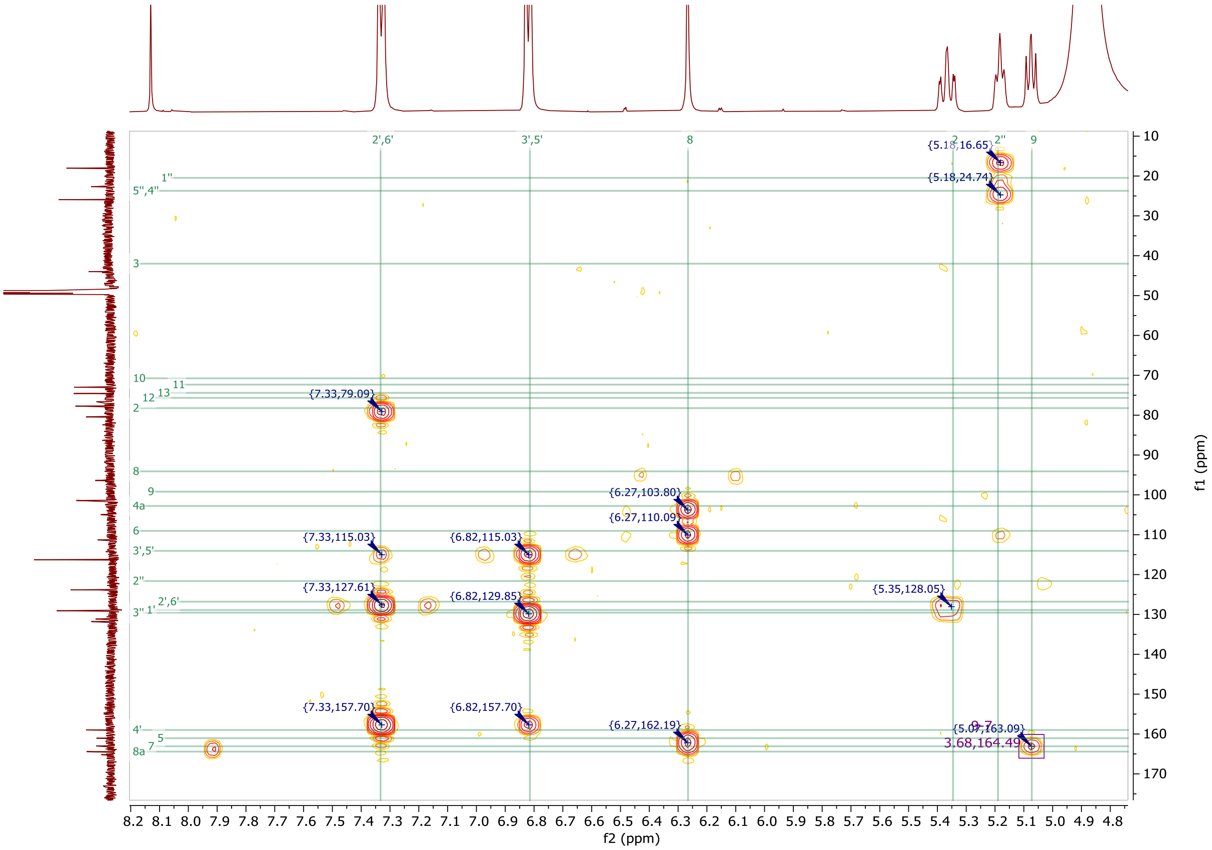

Figure S42: HMBC of 6-PN-7-O-glc

Indications that the glucuronide is attached to the 7 position is that there is a correlation between the anomeric proton of the glucuronide and the C7 as indicated in figure 42

Biosynthesised compounds

XN-4'OH-Glc and XN-7-OH-Glc

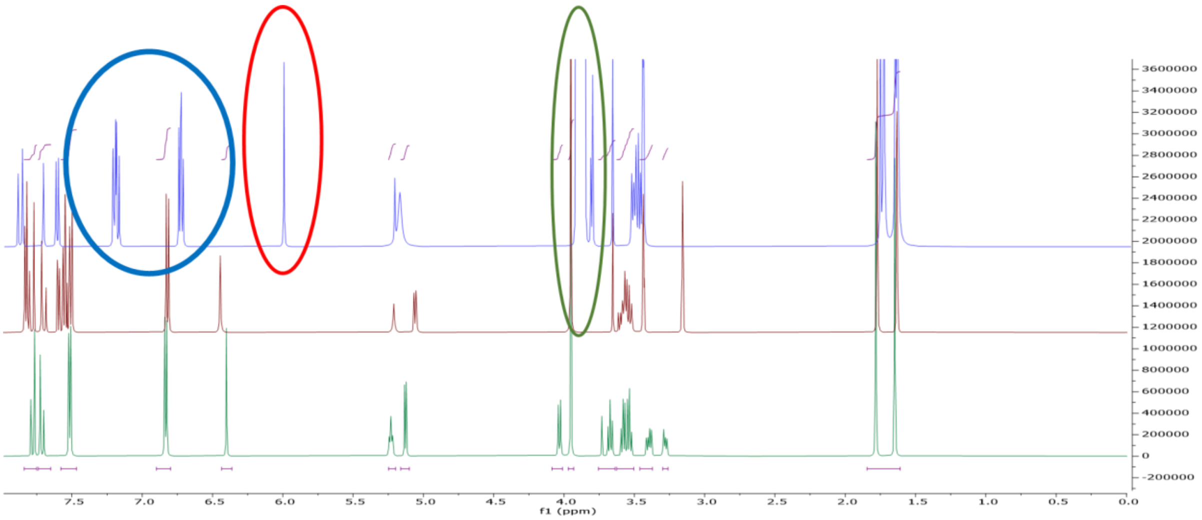

Figure S43:Green is synthesised XN-7-O-glc, Maroon is Biosynthesised XN-7-O-glc and, blue is bio 4'OH-glc

The NMR spectra of the biosynthesised compounds were contaminated and had extremely low abundance therefore, peaks were annotated and all artifacts and contaminated/solvent peaks were removed. An overlay of the H spectra are shown in figure 43 which shows that green and brown are similar, but key changes are in the blue spectra, i.e. the aromatic signals are shifted to lower frequencies and the singlet at position 6 is shifted to lower frequencies when the Glc is conjugated to the phenol.

IXN-C-Glc

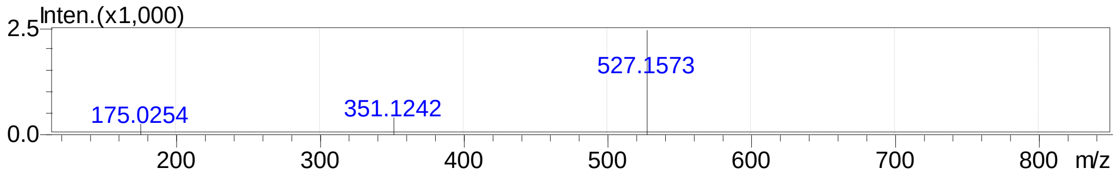

Figure S44: The fragmentation of IXN-C-Glc in DDA

mode *XN-C-Glc*

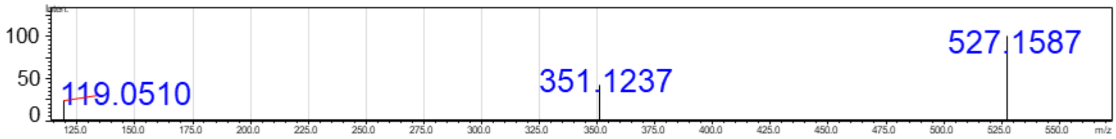

Figure S45: The fragmentation of IXN-C-Glc in DDA mode

2. Enzymatic digestions

XN-7-O-Glc

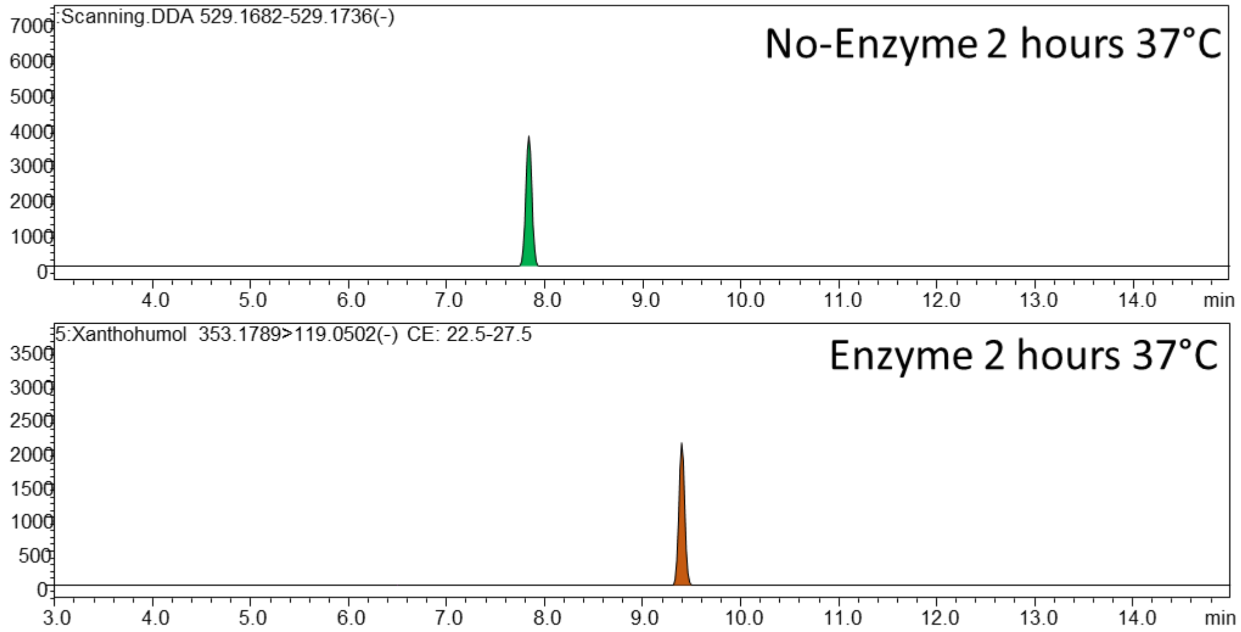

Figure S46: An LC-MS/MS chromatogram of XN-7-O-Glc after enzymatic digestion.

8-PN-7-O-Glc

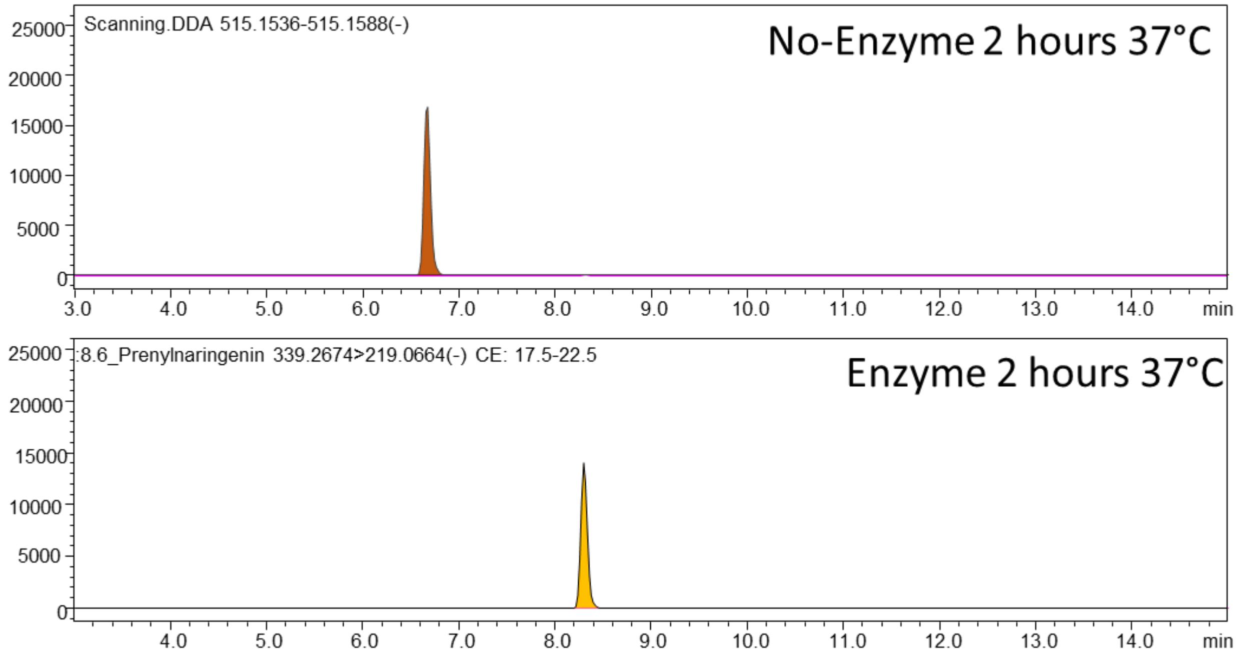

Figure S47: An LC-MS/MS chromatogram of 8PN-7-O-Glc after enzymatic digestion.

6-PN-7-O-Glc

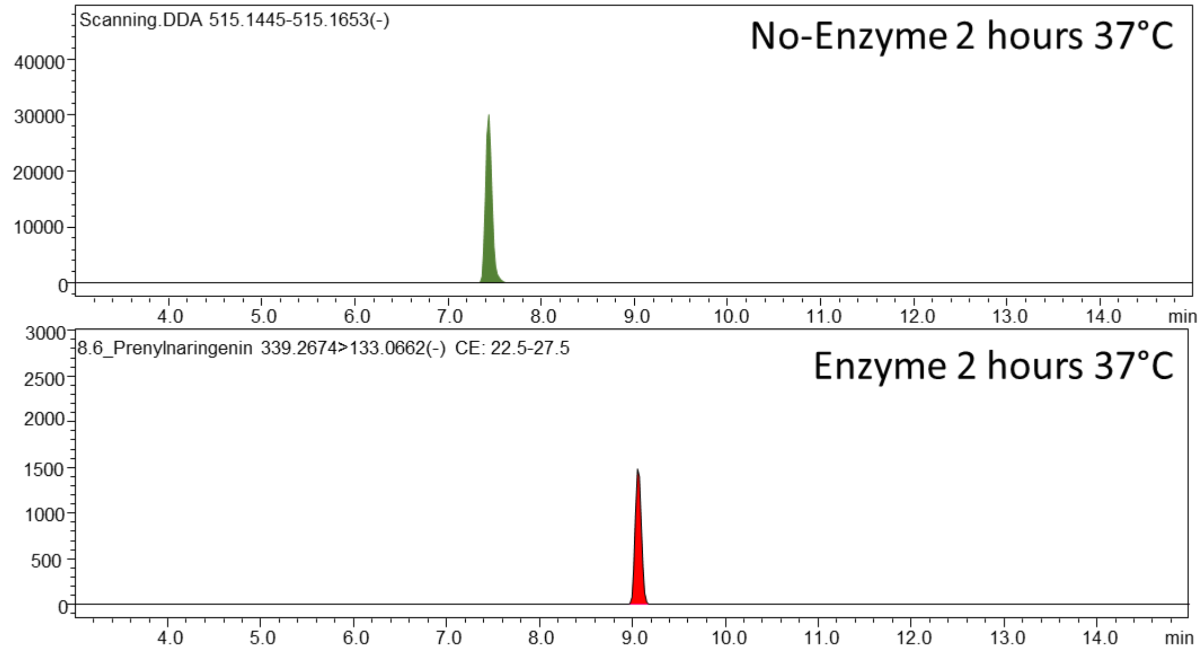

Figure S48: An LC-MS/MS chromatogram of 6PN-7-O-Glc after enzymatic

digestion.

IXN-7-O-GLc

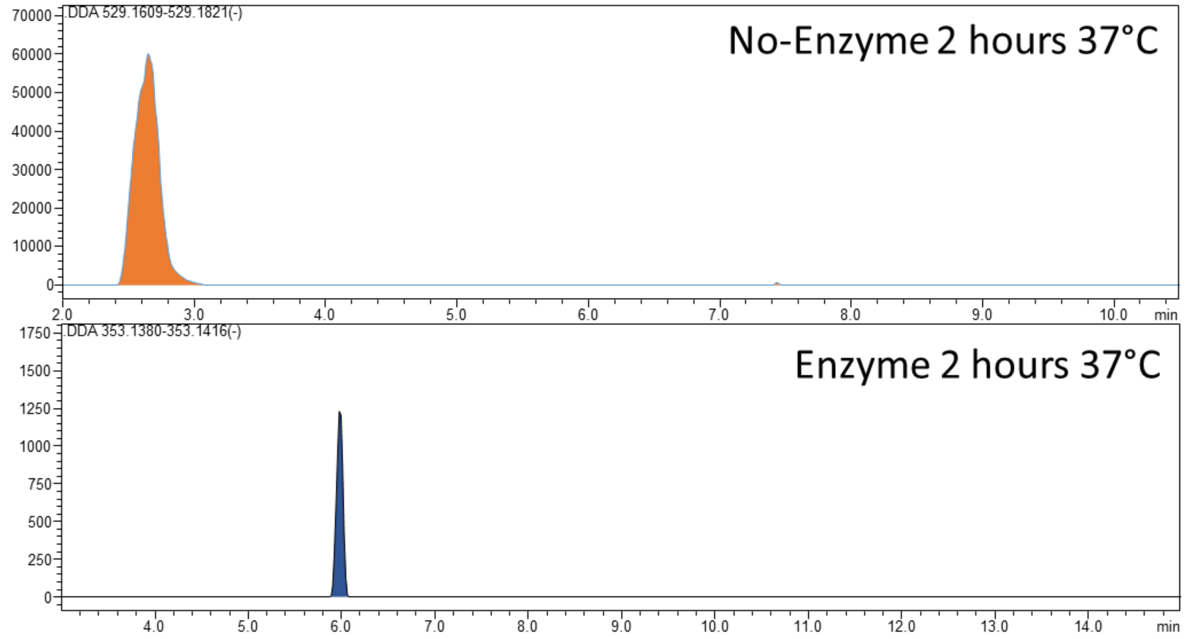

Figure S49: An LC-MS/MS chromatogram of IXN-7-O-Glc after enzymatic digestion. An earlier chromatogram was used where a steeper gradient was used, this was changed in the final runs of the blood hence the earlier elution.

XN-C-4’-O-GLc

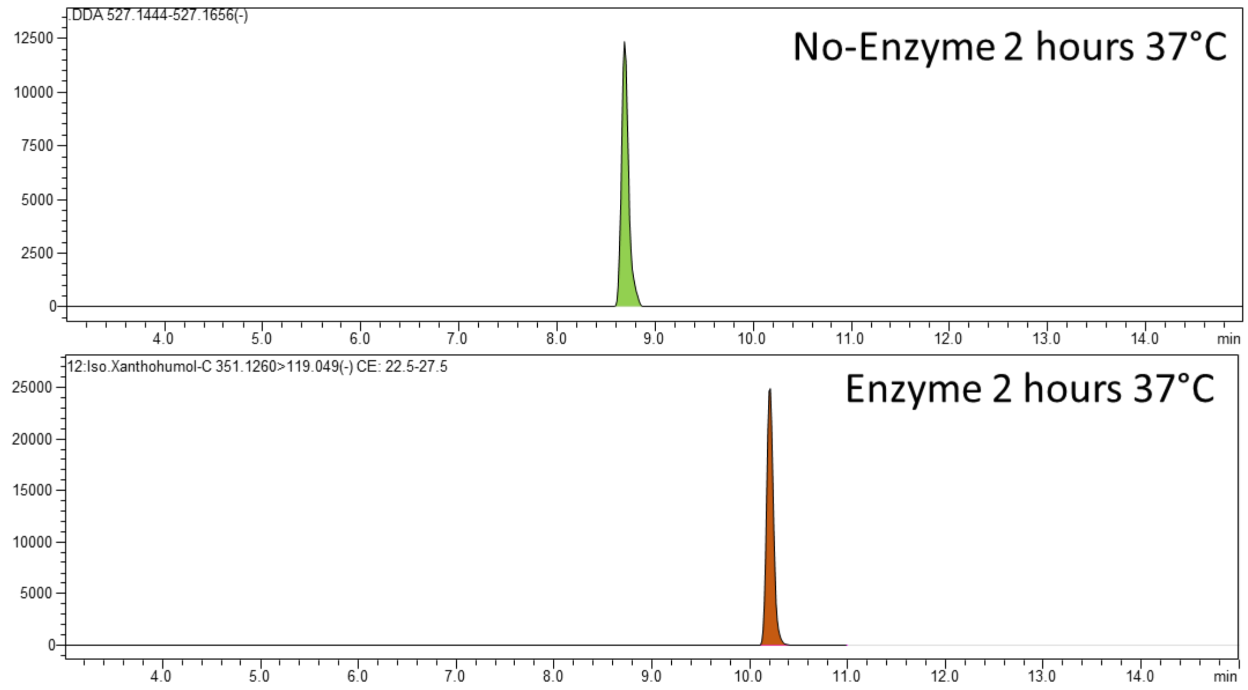

Figure S50: An LC-MS/MS chromatogram of XN-C-4'-O-Glc after enzymatic digestion. IXN-C-4'O-Glc

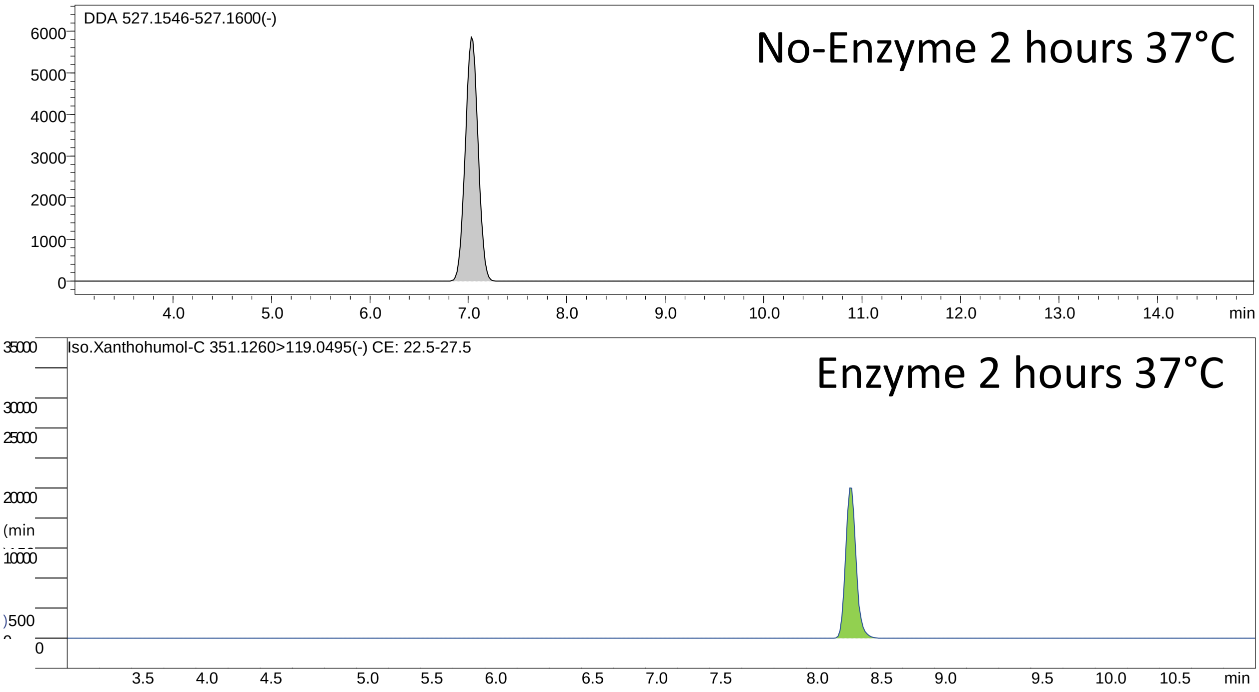

Figure S51: An LC-MS/MS chromatogram of IXN-C-4'-O-Glc after enzymatic digestion.

3. LC-MS/MS of hydroxylated compounds

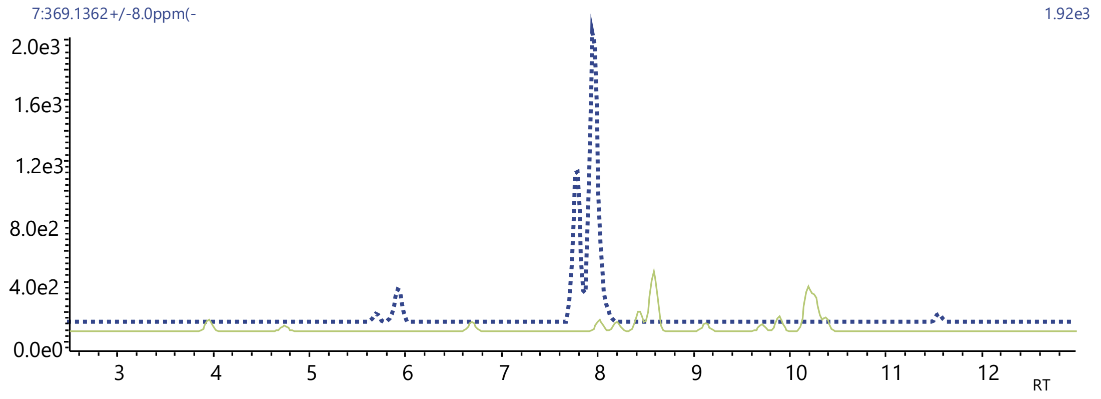

Figure S52: Compounds 5,6 and 7 (blue) overlaid with the analysis of the blood sample (green). No significant peaks were found.

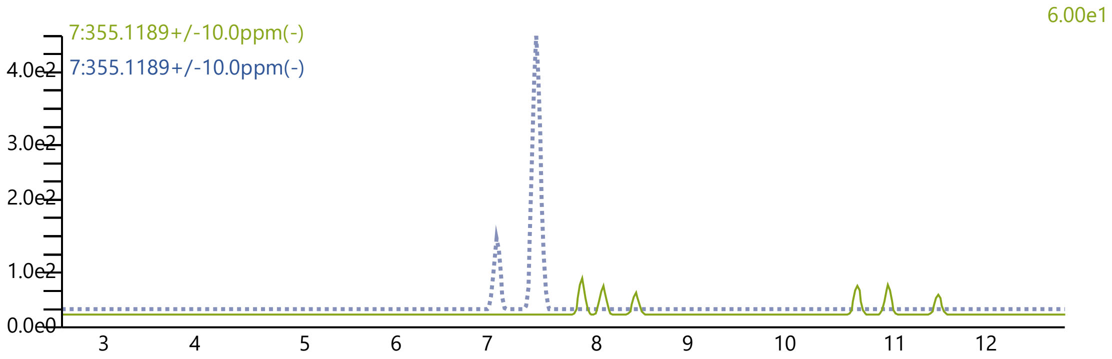

Figure S53: Compounds 8 and 9 (blue) overlaid with the analysis of the blood sample (green). No significant peaks were found.

4. LogP values of the synthesised compounds

Table S1: LogP values of the non-metabolites and the newly synthesised compounds

Non-metabolites	***Xanthohumol***	***Isoxanthohumol***	***8-Prenylnaringenin***	***6-Prenylnaringenin***
LogP	3.91	3.49	3.22	3.22
Hydroxyls	**XN-OH (*Z*) and (*E*)**	**IXN-OH (*E*)**	**8-PN-OH (*E*)**	**6-PN-OH (*E*)**
LogP	2.82	2.43	2.16	2.16
Sulfates	**XN-4'-O-sulfate**	**IXN-4'-O-sulfate**	**8-PN-4'O-sulfate**	**6-PN-7-O-sulfate**
LogP	1.92	1.26	0.74	0.74
Glucuronides	**XN-7-O-glc**	**IXN-7-O-glc**	**8-PN-O-7-glc**	**6-PN-O-7-glc**
LogP	2	1.61	1.34	1.34

References

1. Jongkees, S. A. K.; Withers, S. G., Glycoside Cleavage by a New Mechanism in Unsaturated Glucuronyl Hydrolases. *Journal of the American Chemical Society* **2011,** *133* (48), 19334-19337.

2. Buckett, L.; Schinko, S.; Urmann, C.; Riepl, H.; Rychlik, M., Stable Isotope Dilution Analysis of the Major Prenylated Flavonoids Found in Beer, Hop Tea, and Hops. *Front. Nutr.* **2020,** *7*, 11.
